# Hippocampal Viral-Mediated Urokinase Plasminogen Activator (uPA) Overexpression Mitigates Stress-Induced Anxiety and Depression in Rats by Increasing Brain-Derived Neurotrophic Factor (BDNF) Levels

**DOI:** 10.3390/biom14121603

**Published:** 2024-12-15

**Authors:** Amine Bahi, Jean-Luc Dreyer

**Affiliations:** 1 Department of Basic Medical Sciences, College of Medicine, Ajman University, Ajman, United Arab Emirates; 2 Center of Medical and Bio-Allied Health Sciences Research, Ajman University, Ajman, United Arab Emirates; 3 College of Medicine & Health Sciences, United Arab Emirates University, Al Ain, United Arab Emirates; 4 Division of Biochemistry, University of Fribourg, 1700 Fribourg, Switzerland

**Keywords:** anxiety, BDNF, depression, social stress, lentiviral vectors, urokinase

## Abstract

Emerging evidence suggests the serine protease, urokinase plasminogen activator (uPA), may play an important role in the modulation of mood and cognitive functions. Also, preliminary evidence indicates that uPA modulates BDNF activity that is known to be involved in the pathogenesis of mood disorders. However, the physiological functions of uPA in specific brain regions for mediating stress-related emotional behaviors remain to be elucidated. Therefore, the aim of this study was to assess the role of ectopic uPA expression on anxiety- and depression-like behaviors following social defeat stress in rats. For this purpose, we inspected the behavioral outcomes following bilateral stereotaxic delivery of uPA-overexpressing lentiviral vectors in the hippocampus using a series of behavioral tests. Results show that hippocampal uPA gain-of-function prevented stress-elicited anxiogenic-like effects, as determined in the marble burying, open field, and elevated plus maze tests, with no alterations in spontaneous locomotor activity. Also, ectopic uPA overexpression resulted in anti-depressant-like effects in the sucrose splash, tail suspension, and forced swim tests. Most importantly, uPA overexpression increased hippocampal BDNF levels, and a strong positive correlation was found using the Pearson test. Moreover, the same correlation analysis revealed a strong negative relationship between uPA mRNA and parameters of anxiety- and depression-like behaviors. Taken together, this work highlights the importance of considering uPA activation and provides new insights into the mechanisms involved in the pathophysiology of stress-elicited mood illnesses, which should help in the development of new approaches to tackle depression and anxiety disorders.

## 1. Introduction

The plasminogen-activating system comprises a complex interplay of proteases, their inhibitors, and receptors, collectively orchestrating the conversion of plasminogen to plasmin, a pivotal enzyme in various physiological processes [1]. Within the intricate tapestry of neural circuitry, this proteolytic system exerts significant influence, boasting key members such as tissue-type plasminogen activator (tPA), urokinase-type plasminogen activator (uPA), plasminogen activator inhibitor-1 (PAI-1), urokinase plasminogen activator receptor (uPA-R), neuroserpin, and plasminogen [2]. Inextricably linked to the regulation of neuronal dynamics, the plasmin system plays a discernible role in various facets of neural function, including neurotransmitter release and synaptic plasticity. For instance, through studies employing in vivo microdialysis in knockout models, Ito and colleagues delineated the importance of the plasmin system in regulating depolarization-evoked dopamine release in the nucleus accumbens, a phenomenon central to depression-related behaviors [3,4,5]. Additionally, plasmin’s capacity to activate matrix metalloproteinases (MMPs) underscores its multifaceted involvement in extracellular matrix remodeling, a process implicated in depression pathophysiology [6,7,8,9].

Central to these intricate interactions is brain-derived neurotrophic factor (BDNF), a pivotal mediator of neuroplasticity and mood regulation. The conversion of pro-BDNF to mature BDNF, a process vital for neuronal survival and synaptic potentiation, hinges upon the proteolytic activity of tPA, thus underscoring the nexus between the plasmin system and neurotrophic signaling [10,11]. It is noteworthy that pro-BDNF and BDNF exert contrasting effects. In fact, pro-BDNF induces neuronal apoptosis and long-term synaptic depression, whereas BDNF promotes neuronal survival, facilitates neurogenesis and dendritic growth, and is crucial for long-term potentiation in the hippocampus [11,12]. While the existing evidence suggests plausible connections between the fibrinolytic system and pro-BDNF maturation, rigorous research efforts are essential to establish a definitive link and uncover the underlying mechanisms involved.

Structurally, uPA is a single-chain 411 amino acids serine protease. Once secreted, the zymogen inactive form, pro-uPA, is cleaved by plasmin and becomes activated in the extracellular environment [13]. A large body of evidence has indicated that uPA plays an important role in many physiological processes such as angiogenesis, tissue regeneration, and immune defense mechanisms. In the adult brain, and through in situ hybridization using 35S-cRNA, Masos and Miskin were able to show that uPA transcripts were predominantly found in the subicular complex, as well as in the parietal and entorhinal cortices. Low levels of uPA mRNA were found in the thalamic nucleus, the basolateral amygdala, and in the dentate gyrus of the hippocampus [14]. At the cellular level, uPA mRNA expression was mainly neuronal [14]. Functionally, it has been reported that uPA is involved in a wide range of brain-related disorders [15] such as social behavior [16], seizure susceptibility [17], spontaneous exploration [18], learning and memory [19], schizophrenia [20], fragile X syndrome [21], autism spectrum disorder [22], neurodegenerative disorders like Alzheimer’s disease, Parkinson’s disease, or multiple sclerosis [23]. Also, there may be a strong association between expression levels of other plasmin system members and the pathophysiology of several mood disorders such as depression and anxiety [11]. In previous studies from our laboratory, we used lentiviral-mediated gene transfer technology to show that uPA is implicated in the rewarding properties of cocaine and amphetamine [24], morphine [25], and ethanol [26]. Yet, little attention has been paid to the potential role of uPA in regulating BDNF-mediated antidepressant- and anxiolytic-like properties.

While research targeting the plasminogen system for psychiatric disorders is emerging, its application to stress-related conditions remains limited. Studies, including our own, have demonstrated uPA’s role in addiction and alcoholism, but its impact on stress-induced emotions in preclinical models is unclear. To address this gap, we conducted experiments to investigate the effects of uPA overexpression in the hippocampus, a key emotion-regulating region, on stress responses in rats. We first investigated the effects of social defeat stress on ex vivo uPA mRNA expression in the hippocampus, nucleus accumbens (Nacc), and dorsal striatum (DS). This analysis aimed to determine potential region-specific alterations in uPA mRNA levels following stress exposure, providing insight into how social defeat stress may differentially affect neural circuits associated with mood regulation and behavior. We then employed viral vectors to achieve ectopic uPA gain-of-function and assess its influence on anxiety- and depression-like behaviors following social defeat stress. Based on our prior research, we hypothesized that hippocampal uPA overexpression would exert an anti-depressant and anxiolytic-like effect, mitigating stress-induced emotional deficits.

## 2. Materials and Methods

### 2.1. Animals

Wistar rats were obtained from the central animal facility of the College of Medicine and Health Sciences. Rats were given food and water ad libitum and housed on a 12/12 h light/dark cycle. For the social defeat procedure, experimental males were 10–12-week-old Wistar rats (~230 g) randomly assigned to control or stressed groups. Residents/aggressors were Sprague Dawley retired breeders (~400 g). For ex vivo uPA mRNA expression, we used a first cohort of animals (SD, n = 7 and SH, n = 7). To examine the effects of uPA overexpression, a second cohort of rats was used (SD, n = 29 and SH, n = 8). All experiments were approved by the Institutional Animal Care and Use Committee.

### 2.2. Chronic Social Stress

Social defeat stress was performed as described in our previous work [26], with minor modifications. Resident male rats were occasionally bred with females to sustain the “defender/dominant”-type behavior required for the social defeat (SD) protocol. Before being included in the study, resident rats were chosen based on their aggression level, determined by their attack latency (less than 2 min) toward an unfamiliar male rat. In brief, the social defeat stress protocol began with a 5 min physical interaction between a resident and intruder rat. Threat and attack behaviors were monitored in the resident rats, while avoidance, fleeing, and defensive/submissive behaviors were assessed in the intruder rats. If the intruder displayed a submissive supine posture for more than 10 s or received 15 attack bites, the interaction was terminated before the 5 min mark, as outlined in previous studies [27,28]. If the resident rat did not attack within the initial 5 min, the interaction phase was extended by an additional 15 min before the intruder was introduced to a different resident rat. After this initial interaction, the rats were separated by a wire mesh barrier for a 24 h period, allowing visual and olfactory communication while preventing physical contact. This 24 h sensory interaction was repeated daily for 10 consecutive days, with the intruder rat introduced to a new resident rat each day. Following the final stress exposure, the socially defeated (SD) rats were individually housed, initiating a period of isolation before undergoing stereotaxic surgery. The unstressed control rats were also single housed (SH) for 10 days before surgery. This protocol, based on previous research [29,30,31,32], has been shown to induce behavioral and physiological changes relevant to anxiety- and depression-like behaviors.

The bodyweight of the rats was monitored through daily weighing. Water and food consumption were manually recorded each day, with the rats being provided with a standard chow diet. To measure food intake, all food fragments found in the cages were collected, and the remaining food pellets were weighed. Water consumption was assessed using customized bottles equipped with water sippers, and the daily intake was calculated by subtracting the final water volume from the initial volume.

### 2.3. Lentivirus Construction and Production

The generation of uPA-expressing lentiviral vectors, used in the current study, was described previously [25]. In brief, uPA cDNA was amplified and reverse-transcribed from the rat brain total RNA using specific primers. The sequenced PCR product was cloned into pTK431 viral transfer vector under a tetracycline inducible CMV promoter (Tet-Off system). Vesicular stomatitis virus G pseudotyped lentiviruses were produced by transient triple co-transfection of HEK293T cells, using calcium phosphate of pTK431, ΔNRF, and pMDG-VSV-G plasmids. Viral particles were purified by ultracentrifugation and quantified as described previously [25]. These lentiviral vectors were used in pilot experiments previously conducted in non-stressed rats to optimize dose/timeframe. Also, these same tools were successfully used in our previous work, and uPA-overexpressing lentiviral vector injection results in increasing uPA levels [24,25,33,34].

### 2.4. Stereotaxic Surgery

For lentiviral infection, the rats were anesthetized using a ketamine/xylazine cocktail (100 and 10 mg/kg, respectively). After the rat lost consciousness, confirmed by checking its reflexes, the head area going from the ears to just in between the eyes was shaved with an electric razor. The rat was then placed in the stereotaxic apparatus, and the ear bars were adjusted so that they showed equal reading on both sides. An anterior–posterior incision (~2 cm) was made on the midline of the scalp from between the eyes to the back of the ears. Bulldog clamps were used to pinch off the skin and to keep the incision open, conjunctive tissue was removed using cotton swabs, and the area was cleaned to expose the skull surface. With a hand driller, burr holes were made and each rat was bilaterally infused with 0.5 µL viral solution using the following coordinates [35]: 1st injection: 4.8 mm posterior to Bregma, ±2.5 mm lateral to the medial suture, 3.5 mm ventral to the skull surface; 2nd injection: 4.8 mm posterior to Bregma, ±5 mm lateral to the medial suture, 6 mm ventral to the skull surface, as in our previous study [26]. These coordinates were chosen because it has been shown that all hippocampal sub-regions are involved in chronic stress-induced anxiety- and depression-like behaviors in male C57BL/6 mice [36]. Sham-operated controls were injected with an empty pTK431 viral vector. After surgery, the animals were left to recover for 10 days with unrestricted access to food and water (supplemented with 0.1% saccharin; *w*/*v*). For the Dox-SD group, doxycycline was mixed with drinking water supplemented with 0.1% saccharin and administered immediately after surgery, continuing throughout the entire behavioral testing period (from day 11 to day 27). This ensured consistent doxycycline exposure during the critical phases of the experimental protocol.

The stress–viral injection combination created four test groups: group 1: Sham-single-housed (Sham-SH, n = 8); group 2: Sham-social defeat (Sham-SD, n = 9); group 3: uPA-social defeat (uPA-SD, n = 10); and group 4: uPA + doxycycline-social defeat (Dox-SD, n = 10). It should be highlighted that in the 4th experimental group, and because we used an inducible Tet-Off system, a doxycycline-induced inhibition of uPA expression (switched off) was anticipated.

### 2.5. Anxiety- and Depression-Like Behaviors Assessment

For all behavioral assessments, the same researcher performed the behavioral tests across all experiments to minimize variability in handling and/or testing practices that might affect the results. The behavioral tests were performed in a brightly lit room that was illuminated with fluorescent overhead bulb lights producing steady illumination within the testing room, as depicted in the experimental timeline (Table 1).

#### 2.5.1. Marble Burying Test (MBT)

The MBT was used to evaluate repetitive and anxiety-like behaviors, as described previously [37,38,39,40,41]. Each rat was first allowed to explore a cage (35 cm × 23 cm × 19 cm) for 10 min without marbles. Subsequently, 25 colored glass marbles (1 cm diameter) were evenly spaced in a 5 × 5 grid on 5 cm-deep sawdust bedding. Rats were observed for 30 min, during which the latency to begin digging/burying, the time spent digging, and the number of marbles buried were recorded. A marble was considered buried if at least 60% of its surface was covered by bedding [42]. This test is commonly used to assess repetitive and anxiety-related behaviors linked to hippocampal function [43].

#### 2.5.2. Open Field (OF) Test

The OFT was conducted to assess novelty-induced locomotor activity and exploratory behavior in an approach–avoidance conflict paradigm, as described previously [44,45,46,47]. The arena (90 cm × 90 cm × 30 cm) had a grid floor, dividing it into equal sections. Each rat was placed in the center of the arena and allowed to explore for 10 min. The arena was divided into two zones: an inner/center exploration zone (~25% of the total area) and an outer “home” zone. The time spent in the center zone, an indicator of anxiolytic-like behavior, was manually recorded. Additional parameters, including the number of fecal boli, frequency of rearing, and grid crossings (with all four paws), were also measured. After each trial, the arena was cleaned with 70% ethanol to remove scent cues from previous rats.

#### 2.5.3. Elevated Plus Maze (EPM) Test

The EPM was used to measure anxiety-like behaviors in an approach–avoidance conflict paradigm [48], as previously described [44,49,50,51,52]. The apparatus consisted of a central platform (10 cm × 10 cm), two open arms (50 cm × 10 cm), and two closed arms (50 cm × 10 cm × 40 cm), elevated 50 cm above the floor. Each rat was placed on the central platform and allowed to explore the maze for 5 min. Time spent in the open arms, an index of anxiety-like behavior, and the number of open and closed arm entries were recorded. Locomotor activity was inferred from closed-arm entries [53,54], while grooming and rearing frequencies were also noted. The maze was cleaned with 70% ethanol between trials to eliminate scent traces.

#### 2.5.4. Sucrose Splash Test (SST)

The SST was used to evaluate grooming behavior as an indicator of self-care and depressive-like phenotypes, following established protocols [55]. Each rat’s dorsal coat was sprayed with 1 mL of a 10% sucrose solution using an atomizer to provoke grooming behavior. Latency to initiate grooming, total grooming duration, and frequency of rearing were recorded over 5 min. The SST is a widely used assay for detecting changes in self-care-related behaviors in rodent models of social stress and depression [56,57,58].

#### 2.5.5. Tail Suspension Test (TST)

The TST was conducted to measure immobility duration as an indicator of depressive-like behavior, following established procedures [59]. Each rat was suspended by its tail using adhesive tape on approximately half its length, with a plywood platform positioned below its forepaws to partially reduce tail load [60,61,62]. During the 6 min test, latency to the first immobility episode and total immobility duration were recorded [63]. The platform was cleaned with 70% ethanol between trials to remove odor cues.

#### 2.5.6. Forced Swim Test (FST)

The FST was performed to assess immobility and active behaviors associated with depressive-like phenotypes [64], following established methods [65,66]. Each rat was placed in a transparent glass cylinder (40 cm height × 25 cm diameter) filled with 24 °C water to a depth of 30 cm, ensuring the hind paws could not touch the bottom [67,68,69,70]. During the 6 min test, latency to immobility, total immobility duration, and climbing frequency were recorded. After the test, the rat was dried and kept in a warm recovery box for 10 min before being returned to its home cage. The water was replaced after each trial.

### 2.6. Tissue Collection, Total RNA Isolation, and Quantitative RT-PCR

Rats were euthanized by rapid decapitation 24 h after the completion of the FST (day 27). It should be emphasized that throughout the tissue processing, RNAse-free conditions were meticulously maintained. The brain was removed from the skull and rinsed in ice-cold DEPC-treated Milli-Q water to eliminate surface blood. It was then placed on a cold metal plate. The brain was separated into right and left hemispheres, and the olfactory bulbs were removed. The frontal cortex was excised immediately after the second cut. The striatum (caudate nucleus) was dissected following the third cut with the assistance of two Dumont No. 5 forceps. To collect the hippocampus, the ventral side of the brain was positioned upward, and the midbrain was removed. The exposed hippocampus was then carefully separated from the cortex using two Dumont No. 5 forceps and immediately placed in ice-cold Trizol [44,63,71,72,73]. Total RNA was then extracted and precipitated using isopropanol. cDNA synthesis was performed using the standard SuperScript III reverse transcriptase procedure, and mRNA expression was analyzed with SyberGreen. The quantification of uPA expression was performed using specific primers with the following thermal cycling conditions: an initial denaturation at 95 °C for 5 min, followed by 40 cycles of denaturation at 94 °C for 15 s, and annealing and extension at 60 °C for 1 min. Cyclophilin mRNA served as an internal control and was subjected to the same cycling parameters. Normalization and relative expression analysis of uPA mRNA were carried out using the 2^−△△Ct^ method, with cyclophilin as the control due to its low variability between samples.

### 2.7. BDNF ELISA Quantification

The Rat mature BDNF ELISA kit was used to quantify protein levels in the other brain’s hemisphere following the manufacturer’s protocol (Cat. No. ab213899; Abcam, Cambridge, UK). In brief, total proteins were isolated from the hippocampal tissue using a lysis buffer containing 50 mM Tris-HCl (pH 7.5), 150 mM NaCl, 1 mM EDTA, 1% NP-40, 0.5% deoxycholic acid, 0.1% SDS, 10 µL/mL protease inhibitor cocktail, and 1 mM PMSF. The supernatants were obtained by centrifuging at 12,000× *g* for 15 min at 4 °C. Following this, 96-well microplates were coated with 100 µL of biotinylated primary antibodies mixed with 100 µL of EIA buffer from the kit, along with 100 µL of standard and sample aliquots. The plates were incubated for 90 min at room temperature, after which the samples were aspirated and washed three times with wash buffer. Streptavidin-horseradish peroxidase conjugate (100 µL) was added to each well and incubated for 45 min at room temperature, followed by additional washing. Finally, 100 µL of substrate solution from the kit was added, and the plates were incubated for 30 min. After stopping the color development with a stop solution, the optical density was measured at 450 nm using a plate reader [74,75,76]. Standards and samples were run in triplicates and BDNF concentrations were expressed in picograms of BDNF per milliliter of wet tissue (pg/mL).

### 2.8. Statistical Analysis

Statistical analysis was performed using IBM^®^ SPSS^®^ Statistics for Windows. The data representing the effects of stress on food and water intake were analyzed using a mixed-design ANOVA with repeated measures to examine the effects of stress (two levels: stress vs. no stress) and time (10 levels: day 1 through day 10) on the dependent variable. Mauchly’s test of sphericity was employed to evaluate the assumption of sphericity for the within-subjects factor of time. When the assumption was violated, degrees of freedom were corrected using the Huynh–Feldt adjustment to ensure accurate interpretation of the results. The data representing the effects of stress and uPA overexpression on anxiety- and depression-like behaviors were analyzed using the non-parametric Kruskal–Wallis test. In cases of a significant main effect, post hoc tests were used for pairwise comparisons. Effect sizes (reported as eta squared η^2^) were calculated for the statistically significant effects when appropriate. A principal component analysis (PCA) was conducted to explore the underlying structure of the behavioral data. The suitability of the data for PCA was assessed prior to analysis using the Kaiser–Meyer–Olkin (KMO) measure of sampling adequacy and Bartlett’s test of sphericity. To refine the focus of the study, Spearman correlation analyses, between uPA or BDNF and behavioral parameters, were conducted using standardized Z scores for the behavioral parameters that loaded most strongly onto Component 1. This analysis was prioritized due to Component 1’s relevance to the study hypotheses, as it accounted for the largest proportion of variance and included key measures of stress- and anxiety-related behaviors. The criterion for statistical significance was set at *p* < 0.05. Figures were generated using GraphPad Prism 5.

## 3. Results

### 3.1. Social Defeat Stress Decreased Ex Vivo Hippocampal uPA Expression

**Figure 1:**
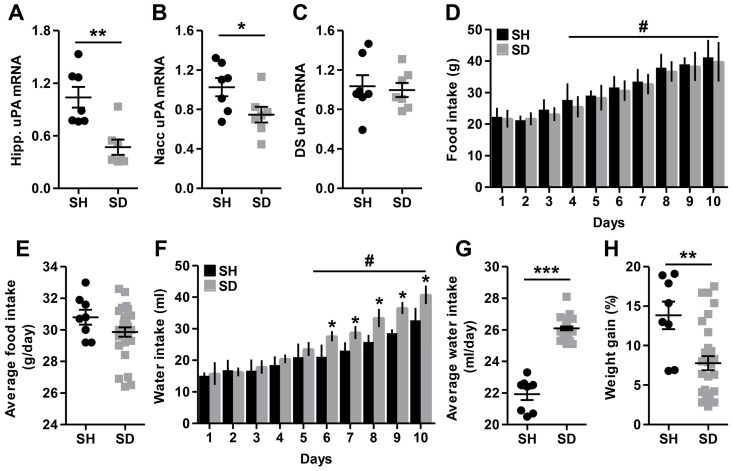
Effects of chronic social defeat on uPA expression and general physiological parameters. Data are presented as means ± SEM for the effects of social defeat on uPA mRNA expression in the (**A**) hippocampus, (**B**) nucleus accumbens, and (**C**) dorsal striatum (SD, n = 7 and SH, n = 7). For physiological parameters the data represent the effects of social defeat on (**D**) daily food intake, (**E**) average food intake, (**F**) daily water intake, (**G**) average food intake, and (**H**) percentage of weight gain (SD, n = 29 and SH, n = 8). * *p* < 0.05, ** *p* < 0.005, and *** *p* < 0.001; # *p* < 0.05 vs. day 1.

In this investigative experiment, using a first cohort of animals, we examined ex vivo uPA mRNA expression by means of RT-PCR in the hippocampus, Nacc, and dorsal striatum after 10 days of stress exposure (SD, n = 7). Control animals were single housed the whole time (SH, n = 7). In the hippocampus, and as depicted in Figure 1A, social defeat stress decreased ex vivo uPA mRNA expression (F_(1,12)_ = 14.954; *p* = 0.002; η^2^ = 0.555). We also tested other emotions-related brain regions and we found that SD decreased ex vivo uPA mRNA expression in the Nacc (F_(1,12)_ = 5.288; *p* = 0.040; η^2^ = 0.306; Figure 1B), but not in the dorsal striatum (F_(1,12)_ = 0.085; *p* = 0.775; η^2^ = 0.007; Figure 1C). These findings indicate that chronic social stress induces a down-regulation of ex vivo uPA mRNA expression in the hippocampus and the Nacc, but not in the dorsal striatum.

### 3.2. Social Defeat Stress Increased Water Intake but Decreased Weight Gain

Using a second cohort of animals, we examined the effects of stress on food and water intake and bodyweight gain in male Wistar rats that were exposed to social defeat (SD, n = 29) for 10 days, with control animals being single housed (SH, n = 8) for the same period of time as described in the Methods section. Consistent with previous findings [77], examination of cumulative food intake showed that SD exposure had no effect on food consumption. The assumptions analysis for a mixed (2 × 10) (stress × time) ANOVA showed non-compliance with sphericity, as the Mauchly’s test was found significant (χ^2^_(44)_ = 108.224; *p* < 0.001). Consequently, the Huynh–Feldt test for the degrees of freedom was used and the analysis indicated a significant effect of time (F_(7.042,246.484)_ = 96.683; *p* < 0.001; η^2^ = 0.734) (Figure 1D). In fact, there was a clear and consistent change in food intake over the 10-day period being investigated. However, the effect of stress (F_(1,35)_ = 2.337; *p* = 0.135; η^2^ = 0.063) and the interaction between the two factors (F_(7.042,246.484)_ = 0.248; *p* = 0.973; η^2^ = 0.007) were not found to be significant. As expected, there was no significant effect of stress on daily average food intake (F_(1,35)_ = 2.302; *p* = 0.138; η^2^ = 0.062) (Figure 1E). We then tested the effects of SD on water intake, and Mauchly’s test showed that the assumption of sphericity had been violated (χ^2^(44) = 66.283; *p* = 0.018). Therefore, a Huynh–Feldt correction was used and the analysis indicated a significant effect of time (F_(7.799,272.950)_ = 208.263; *p* < 0.001; η^2^ = 0.856) (Figure 1F). Interestingly, daily water intake increased with social defeat (F_(1,35)_ = 98.2761; *p* < 0.001; η^2^ = 0.850), with significant interaction (F_(7.799,272.950)_ = 11.133; *p* < 0.001; η^2^ = 0.241). As expected, there was a significant effect of stress on daily average water intake, which was markedly increased in socially defeated rats (F_(1,35)_ = 198.276; *p* < 0.001; η^2^ = 0.850) (Figure 1G). Finally, we assessed the effects of stress exposure on bodyweight gain and the analysis revealed that in terms of percent weight gain, SD animals gained less weight than SH controls (F_(1,35)_ = 9.974; *p* = 0.003; η^2^ = 0.222) (Figure 1H). Similarly, absolute weight gain was significantly lower in social defeat-exposed rats (SD) compared to single-housed controls (SH) (F_(1,35)_ = 10.401; *p* = 0.003; η^2^ = 0.229). While the data suggest a trend toward reduced bodyweight in the chronically stressed group compared to the control group, the conclusion is limited by the difference in sample sizes between the groups.

### 3.3. uPA Overexpression Counteracted Social Stress-Elicited Mood Disorders

Next, we assessed the impact of ectopic hippocampal uPA overexpression on SD-induced anxiogenic-like effects in experimental Wistar rats. For this purpose, SH rats were stereotaxically injected with Sham control vectors (n = 8). However, the SD cohort (n = 29) was divided into three groups as follows: Sham vectors (Sham-SD; n = 9); uPA (uPA-SD; n = 10); and uPA with rats having access to doxycycline in drinking water to block transgene expression (Dox-SD; n = 10).

#### 3.3.1. Marble Burying Test (MBT)

**Figure 2:**
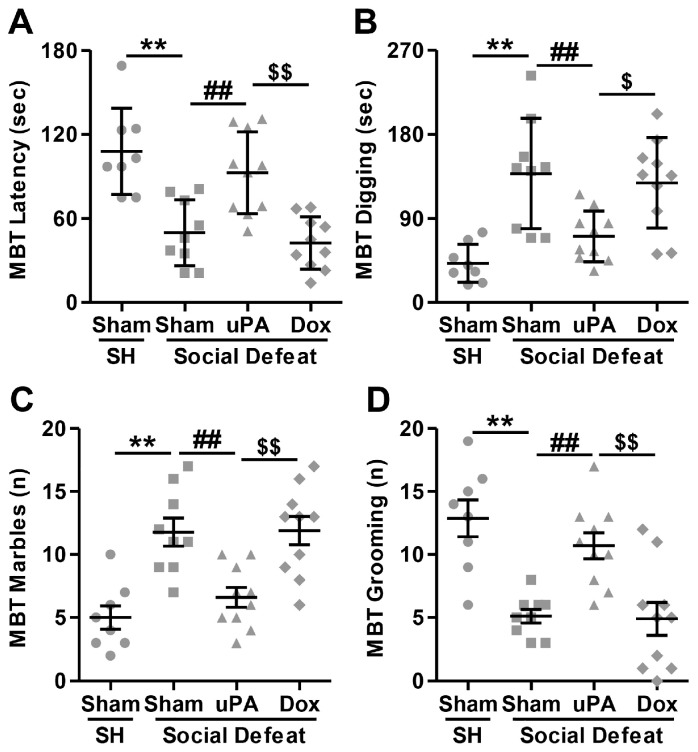
Effects of lentiviral-mediated uPA overexpression on chronic social defeat-induced anxiety-like behavior in the MBT. Data are presented as individual data points with means ± SEM. (**A**) Latency to start digging, (**B**) total time spent digging, (**C**) number of buried marbles, and (**D**) number of grooming episodes. ** *p* < 0.001 for Sham-SH vs. Sham-SD; ## *p* < 0.01 for Sham-SD vs. uPA-SD; $$ *p* < 0.01 and $ *p* < 0.05 for uPA-SD vs. Dox-SD. Sham-SH, n = 8; Sham-SD, n = 9; uPA-SD, n = 10; Dox-SD, n = 10.

We assessed the role of uPA using simple, hippocampus-dependent tasks, including latency and total digging duration, marble burying, and grooming [78]. For this purpose, the first experiment tested whether uPA overexpression would alter time spent digging and the number of buried marbles compared with single-housed rats that had undergone Sham surgery. For the latency to start digging, the Kruskal–Wallis test was used and it was found significant (H(3) = 20.955; *p* < 0.001). As depicted in Figure 2A, post hoc evaluations indicated that Sham-injected rats exposed to social defeat stress exhibited a significant decrease in the latency to start digging compared to single-housed animals (Sham-SH vs. Sham-SD; *p* = 0.002). Interestingly, uPA overexpression in the hippocampus produced differential effects on this behavior depending on the presence or absence of doxycycline. Specifically, in the absence of doxycycline, uPA-overexpressing rats displayed a significant increase in the latency to start digging compared to both Sham-injected (Sham-SD vs. uPA-SD; *p* = 0.011) and uPA-overexpressing rats treated with doxycycline (uPA-SD vs. Dox-SD; *p* = 0.001). However, no significant difference in digging latency was observed between the uPA-overexpressing rats treated with doxycycline and the Sham-injected stressed rats (Sham-SD vs. Dox-SD; *p* = 0.528). These findings suggest that uPA overexpression, in the absence of doxycycline, may reduce compulsive-like behavior following social stress exposure, while doxycycline treatment appears to mitigate this effect. For the time spent digging, the Kruskal–Wallis test was found significant (H(3) = 19.483; *p* < 0.001). As shown in Figure 2B, the analysis revealed an inverse pattern compared to the latency to start digging. In fact, Sham-injected rats exposed to social stress showed a significant increase in the total time spent digging compared to control animals (Sham-SH vs. Sham-SD; *p* < 0.001), indicating heightened compulsive-like behavior under stress. However, uPA overexpression in the hippocampus led to a notable decrease in total digging time in the absence of doxycycline, with uPA-overexpressing rats spending significantly less time digging compared to both Sham-injected (Sham-SD vs. uPA-SD; *p* = 0.018) and uPA-overexpressing rats treated with doxycycline (uPA-SD vs. Dox-SD; *p* = 0.034). In contrast, no significant difference in total digging time was observed between uPA-overexpressing rats treated with doxycycline and Sham-injected stressed rats (Sham-SD vs. Dox-SD; *p* = 0.765). For the number of buried marbles, a significant group effect was found (H(3) = 19.343; *p* < 0.001). As shown in Figure 2C, pairwise comparisons indicated that Sham-injected rats exposed to social stress buried significantly more marbles compared to their unstressed counterparts (Sham-SH vs. Sham-SD; *p* < 0.001), indicating increased compulsive-like behavior in response to stress. However, uPA overexpression in the hippocampus led to a significant decrease in the number of buried marbles in the absence of doxycycline, with uPA-overexpressing rats burying fewer marbles compared to both Sham (Sham-SD vs. uPA-SD; *p* = 0.007) and uPA-overexpressing rats treated with doxycycline (uPA-SD vs. Dox-SD; *p* = 0.006). In contrast, there was no significant difference in the number of marbles buried between uPA-overexpressing rats treated with doxycycline and Sham-injected stressed rats (Sham-SD vs. Dox-SD; *p* = 0.999). Finally, for the number of grooming episodes/frequency, a significant group effect was found (H(3) = 20.129; *p* < 0.001). As depicted in Figure 2D, post hoc analyses showed that Sham-injected rats exposed to social defeat stress displayed a significant decrease in grooming episodes compared to their single-housed counterparts (Sham-SH vs. Sham-SD; *p* < 0.001), indicating reduced grooming behavior following social stress exposure. In contrast, uPA overexpression in the hippocampus increased grooming frequency in the absence of doxycycline, with uPA-overexpressing rats engaging in significantly more grooming episodes than both Sham-injected (Sham-SD vs. uPA-SD; *p* = 0.005) and uPA-overexpressing rats treated with doxycycline (uPA-SD vs. Dox-SD; *p* = 0.004). However, there was no significant difference in grooming frequency between uPA-overexpressing rats treated with doxycycline and Sham-injected, stressed rats (Sham-SD vs. Dox-SD; *p* = 0.994).

#### 3.3.2. Open Field (OF) Test

**Figure 3:**
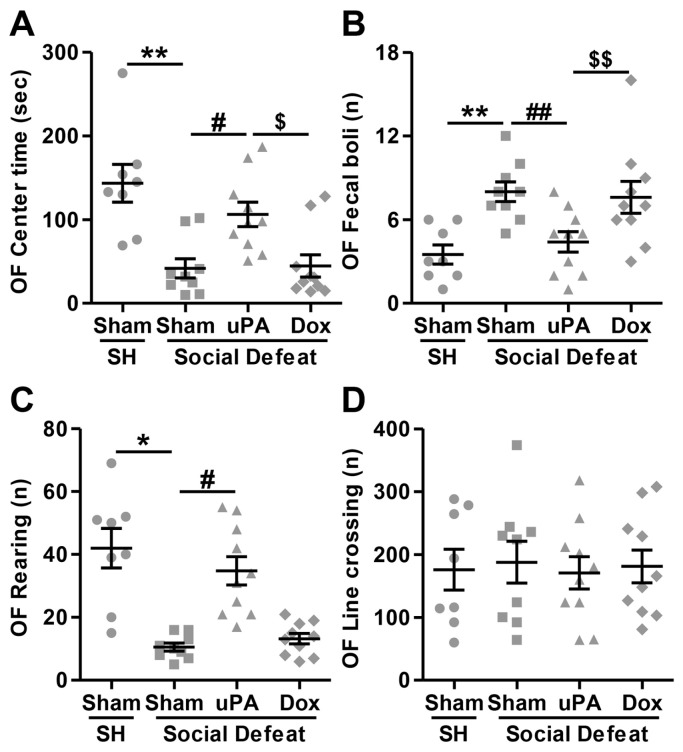
Effects of lentiviral-mediated uPA overexpression on chronic social defeat-induced anxiety-like behavior in the OF test. Data are presented as individual data points with means ± SEM. (**A**) Time spent in the center of the arena, (**B**) number of fecal boli, (**C**) number of rearing episodes, and (**D**) number of line crossings. ** *p* < 0.001 and * *p* < 0.005 for Sham-SH vs. Sham-SD; ## *p* < 0.01 and # *p* < 0.05 for Sham-SD vs. uPA-SD; $$ *p* < 0.01 and $ *p* < 0.05 for uPA-SD vs. Dox-SD. Sham-SH, n = 8; Sham-SD, n = 9; uPA-SD, n = 10; Dox-SD, n = 10.

The non-parametric Kruskal–Wallis test revealed a significant main effect for the time spent in the center of the arena across experimental groups (H(3) = 18.344; *p* < 0.001), indicating differences in anxiety-like behavior. As shown in Figure 3A, post hoc analyses showed that Sham-injected rats exposed to social defeat stress spent significantly less time in the center compared to single-housed controls (Sham-SH vs. Sham-SD; *p* < 0.001), reflecting increased anxiety-like behavior following stress exposure. In contrast, uPA overexpression in the hippocampus, in the absence of doxycycline, led to a significant increase in time spent in the center compared to Sham-injected stressed animals (Sham-SD vs. uPA-SD; *p* = 0.010) and uPA-overexpressing rats treated with doxycycline (uPA-SD vs. Dox-SD; *p* = 0.009). However, no significant difference was observed between uPA-overexpressing rats treated with doxycycline and Sham-injected stressed rats (Sham-SD vs. Dox-SD; *p* = 0.954). For the number of fecal boli, the non-parametric Kruskal–Wallis test was found significant across groups (H(3) = 15.809; *p* = 0.001), indicating variations in anxiety-related responses. As depicted in Figure 3B, pairwise comparisons indicated that Sham-injected rats exposed to social defeat stress produced significantly more fecal boli compared to single-housed rats (Sham-SH vs. Sham-SD; *p* < 0.001), suggesting elevated anxiety. uPA overexpression in the absence of doxycycline significantly reduced the number of fecal boli (Sham-SD vs. uPA-SD; *p* = 0.005), while this reduction was reversed by doxycycline treatment (uPA-SD vs. Dox-SD; *p* = 0.026). There was no significant difference between uPA-overexpressing rats treated with doxycycline and Sham-injected stressed rats (Sham-SD vs. Dox-SD; *p* = 0.533). For the number of rearing episodes, the non-parametric Kruskal–Wallis test was found significant across groups (H(3) = 26.326; *p* < 0.001), indicating differences in exploratory behavior. As displayed in Figure 3C, post hoc analyses demonstrated that Sham-injected stressed rats displayed significantly fewer rearing episodes compared to single-housed controls (Sham-SH vs. Sham-SD; *p* < 0.001), suggesting reduced exploratory behavior under stress conditions. However, uPA overexpression in the absence of doxycycline significantly increased the number of rearing episodes (Sham-SD vs. uPA-SD; *p* = 0.001), an effect that was reversed by doxycycline treatment (uPA-SD vs. Dox-SD; *p* = 0.002). No significant difference was observed between uPA-overexpressing rats treated with doxycycline and Sham-injected stressed rats (Sham-SD vs. Dox-SD; *p* = 0.497). Finally, and as shown in Figure 3D, the non-parametric Kruskal–Wallis test revealed no significant main effect for the number of line crossings across experimental groups (H(3) = 0.121; *p* = 0.989), suggesting that general locomotor activity was not significantly impacted by either social defeat stress or uPA overexpression. Consequently, no post hoc evaluations were performed for this measure.

#### 3.3.3. Elevated Plus Maze (EMP) Test

**Figure 4:**
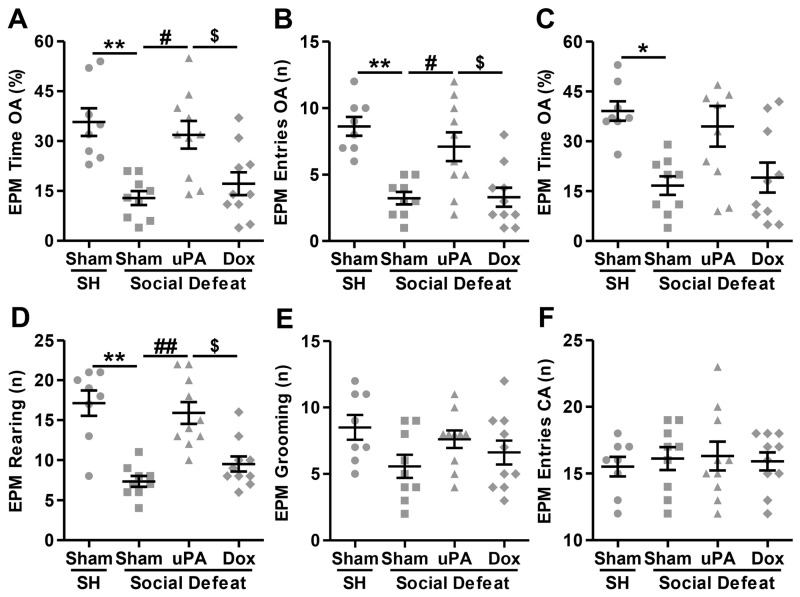
Effects of lentiviral-mediated uPA overexpression on chronic social defeat-induced anxiety-like behavior in the EPM test. Data are presented as individual data points with means ± SEM. (**A**) Percentage of time spent in the OAs, (**B**) number of entries into the OAs, (**C**) percentage of entries into the OAs, (**D**) number of rearing episodes, (**E**) number of grooming episodes, and (**F**) number of entries into the CAs. ** *p* < 0.001 and * *p* < 0.05 for Sham-SH vs. Sham-SD; ## *p* < 0.001 and # *p* < 0.05 for Sham-SD vs. uPA-SD; $ *p* < 0.05 for uPA-SD vs. Dox-SD. Sham-SH, n = 8; Sham-SD, n = 9; uPA-SD, n = 10; Dox-SD, n = 10.

In the EPM test, the statistical analysis revealed significant main effects for several behavioral measures, indicating differences between groups in anxiety-like behavior and exploratory activity. In detail, the Kruskal–Wallis test revealed a significant main effect for time spent in the open arms (H(3) = 17.827; *p* < 0.001). As depicted in Figure 4A, post hoc analyses showed that Sham-injected rats exposed to social defeat stress spent significantly less time in the OAs compared to single-housed controls (Sham-SH vs. Sham-SD; *p* < 0.001), indicating an anxiogenic-like response. However, hippocampal uPA overexpression, in the absence of doxycycline, significantly increased the time spent in the OAs compared to Sham-injected stressed rats (Sham-SD vs. uPA-SD; *p* = 0.002) an effect that was abrogated by doxycycline treatment (uPA-SD vs. Dox-SD; *p* = 0.013). There was no significant difference between uPA-overexpressing rats treated with doxycycline and Sham-injected stressed rats (Sham-SD vs. Dox-SD; *p* = 0.508). For the number of entries into the OAs, the statistical analysis indicated a significant difference across groups arms (H(3) = 18.241; *p* < 0.001). As displayed in Figure 4B, pairwise comparisons revealed that Sham-injected stressed rats made significantly fewer entries into the OAs compared to single-housed rats (Sham-SH vs. Sham-SD; *p* < 0.001). However, uPA overexpression significantly increased the number of entries into the OAs without (Sham-SD vs. uPA-SD; *p* = 0.013) but not with doxycycline treatment (uPA-SD vs. Dox-SD; *p* = 0.009), while no significant difference was observed between uPA-overexpressing rats treated with doxycycline and Sham-injected stressed rats (Sham-SD vs. Dox-SD; *p* = 0.952). Similarly, the Kruskal–Wallis test showed a significant effect for the percentage of entries into the OAs (H(3) = 13.309; *p* = 0.004). As shown in Figure 4C, post hoc analyses revealed that Sham-injected stressed animals had a significantly lower percentage of entries into the OAs of the maze compared to single-housed controls (Sham-SH vs. Sham-SD; *p* = 0.003). However, hippocampal uPA overexpression significantly increased the percentage of entries into the OAs in the absence (Sham-SD vs. uPA-SD; *p* = 0.021) but not in the presence of doxycycline (uPA-SD vs. Dox-SD; *p* = 0.032). There was no significant difference between uPA-overexpressing rats treated with doxycycline and Sham-injected stressed rats (Sham-SD vs. Dox-SD; *p* = 0.813). For the number of rearing episodes/frequency, the Kruskal–Wallis test showed a significant effect across groups (H(3) = 22.011; *p* < 0.001). As depicted in Figure 4D, pairwise comparisons indicated that Sham-injected stressed animals displayed significantly fewer rearing episodes compared to single-housed controls (Sham-SH vs. Sham-SD; *p* < 0.001), reflecting reduced exploratory behavior. However, uPA overexpression significantly increased the number of rearing episodes (Sham-SD vs. uPA-SD; *p* < 0.001), an effect that was reversed by doxycycline treatment (uPA-SD vs. Dox-SD; *p* = 0.007), while no significant difference was observed between uPA-overexpressing rats treated with doxycycline and Sham-injected stressed rats (Sham-SD vs. Dox-SD; *p* = 0.251). Finally, the Kruskal–Wallis test did not reveal any significant differences between groups for the number of grooming episodes (H(3) = 5.055; *p* = 0.168; Figure 4E) and the number of entries into the CAs (H(3) = 0.383; *p* = 0.944; Figure 4F). Therefore, no post hoc tests were performed for these measures.

#### 3.3.4. Sucrose Splash Test (SST)

**Figure 5:**
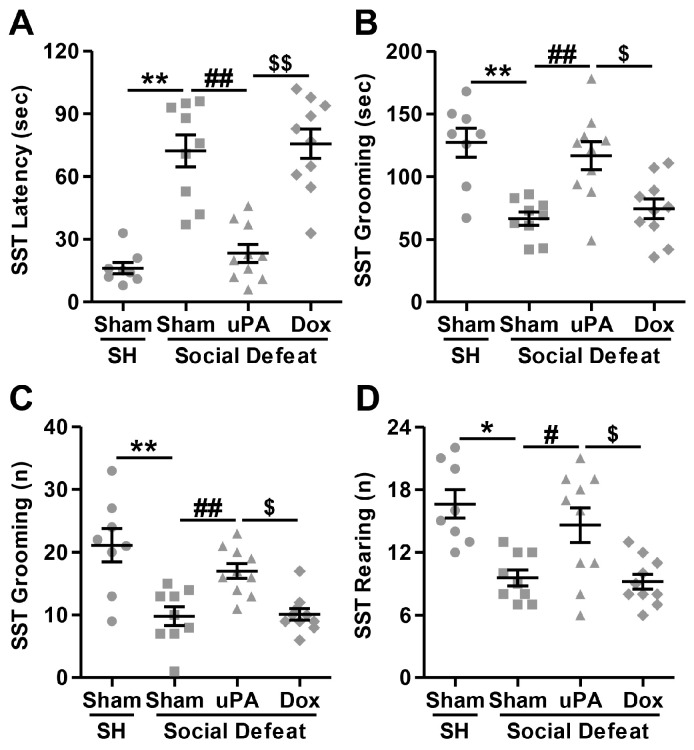
Effects of lentiviral-mediated uPA overexpression on chronic social defeat-induced depression-like behavior in the SST. Data are presented as individual data points with means ± SEM. (**A**) Latency to start grooming, (**B**) total time spent grooming, (**C**) number of grooming episodes, and (**D**) number of rearing episodes. ** *p* < 0.001 and * *p* < 0.05 for Sham-SH vs. Sham-SD; ## *p* < 0.001 and # *p* < 0.05 for Sham-SD vs. uPA-SD; $$ *p* < 0.001 and $ *p* < 0.05 for uPA-SD vs. Dox-SD. Sham-SH, n = 8; Sham-SD, n = 9; uPA-SD, n = 10; Dox-SD, n = 10.

In the SST, we investigated the association between fur coat alteration and grooming behavior following social defeat stress exposure and uPA overexpression, and the results are shown in Figure 5. For grooming latency, the Kruskal–Wallis test revealed a significant main effect across groups (H(3) = 25.397; *p* < 0.001). As displayed in Figure 5A, post hoc analyses indicated that Sham-injected rats exposed to social defeat stress had a significantly longer latency to start grooming compared to single-housed controls (Sham-SH vs. Sham-SD; *p* < 0.001), indicating stress-induced reductions in self-care behavior. However, hippocampal uPA overexpression significantly reduced the latency to start grooming compared to Sham-injected stressed rats (Sham-SD vs. uPA-SD; *p* = 0.002), reversing the stress effect in the absence of doxycycline. However, when doxycycline was administered to uPA-overexpressing rats, the latency to start grooming was significantly abrogated (uPA-SD vs. Dox-SD; *p* < 0.001) with no significant differences observed between uPA-overexpressing rats treated with doxycycline and Sham-injected stressed rats (Sham-SD vs. Dox-SD; *p* = 0.826), indicating that doxycycline treatment abrogated the protective effect of uPA. As expected, the statistical analysis revealed a significant difference in grooming duration across groups (H(3) = 17.748; *p* < 0.001). As shown in Figure 5B, post hoc evaluations indicated that stressed Sham-injected rats groomed for a significantly shorter time compared to single-housed controls (Sham-SH vs. Sham-SD; *p* < 0.001), again reflecting reduced self-care behavior under stress. uPA overexpression without doxycycline treatment significantly increased grooming duration in stressed rats (Sham-SD vs. uPA-SD; *p* = 0.003), suggesting that uPA restored grooming behavior. However, the addition of doxycycline nullified this effect (uPA-SD vs. Dox-SD; *p* = 0.013), as uPA-overexpressing rats treated with doxycycline exhibited grooming times similar to Sham-injected stressed animals (Sham-SD vs. Dox-SD; *p* = 0.579). In addition, the Kruskal–Wallis test showed a significant main effect for grooming frequency (H(3) = 18.597; *p* < 0.001). As depicted in Figure 5C, pairwise comparisons revealed that Sham-injected stressed rats groomed significantly less frequently than single-housed controls (Sham-SH vs. Sham-SD; *p* = 0.001), suggesting stress-induced reductions in self-care. uPA overexpression, in the absence of doxycycline, significantly increased grooming frequency compared to Sham-injected stressed rats (Sham-SD vs. uPA-SD; *p* = 0.007), demonstrating a reversal of the stress effect. Doxycycline treatment, however, abolished the uPA effect (uPA-SD vs. Dox-SD; *p* = 0.004), as grooming frequency in uPA-overexpressing rats with doxycycline was not significantly different from Sham-injected stressed rats (Sham-SD vs. Dox-SD; *p* = 0.922). Finally, the Kruskal–Wallis test indicated a significant difference in rearing frequency across the groups (H(3) = 15.690, *p* = 0.001). As depicted in Figure 5D, post hoc analyses revealed that Sham-injected stressed rats exhibited significantly fewer rearing episodes than single-housed controls (Sham-SH vs. Sham-SD; *p* = 0.002), reflecting decreased exploratory activity. However, hippocampal uPA overexpression in the absence of doxycycline significantly increased rearing frequency (Sham-SD vs. uPA-SD; *p* = 0.036), counteracting the stress-induced reduction. However, this effect was not observed in uPA-overexpressing rats treated with doxycycline (uPA-SD vs. Dox-SD; *p* = 0.018), where rearing frequency remained comparable to Sham-injected stressed rats (Sham-SD vs. Dox-SD; *p* = 0.832).

#### 3.3.5. Tail Suspension Test (TST)

**Figure 6:**
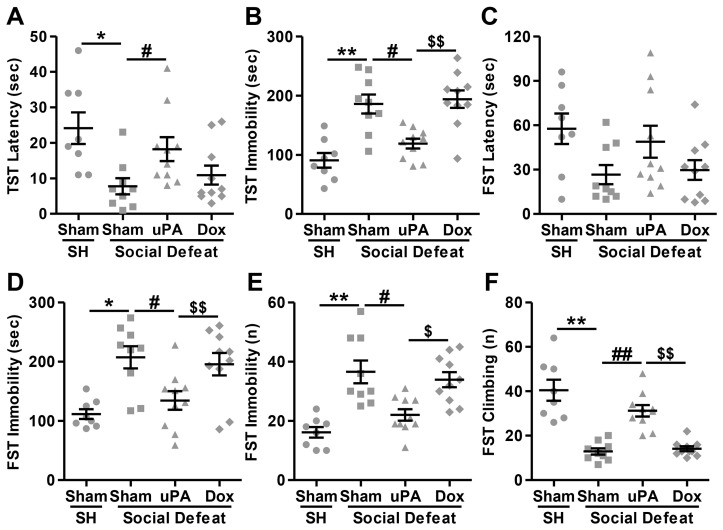
Effects of lentiviral-mediated uPA overexpression on chronic social defeat-induced depression-like behavior in the TST and FST. Data are presented as individual data points with means ± SEM. For the TST, the data represent the (**A**) latency to the first immobility episode and (**B**) total duration of immobility. For the FST, the data represent the (**C**) latency to the first immobility episode, (**D**) total duration of immobility, (**E**) number of immobility episodes, and (**F**) number of climbing episodes. ** *p* < 0.001 and * *p* < 0.05 for Sham-SH vs. Sham-SD; ## *p* < 0.001 and # *p* < 0.05 for Sham-SD vs. uPA-SD; $$ *p* < 0.001 and $ *p* < 0.05 for uPA-SD vs. Dox-SD. Sham-SH, n = 8; Sham-SD, n = 9; uPA-SD, n = 10; Dox-SD, n = 10.

Lastly, we investigated the association between coping strategies and behavioral despair following social defeat stress exposure and uPA overexpression, and the results are shown in Figure 6. The Kruskal–Wallis test revealed a significant main effect for latency to the first immobility episode (H(3) = 15.711, *p* = 0.001). As depicted in Figure 6A, post hoc analyses indicated that Sham-injected rats exposed to social defeat stress exhibited a significantly shorter latency to the first immobility episode compared to single-housed controls (Sham-SH vs. Sham-SD; *p* = 0.001), suggesting that social stress induced an increased susceptibility to behavioral despair. However, hippocampal uPA overexpression in the absence of doxycycline significantly prolonged the latency to the first immobility episode in stressed animals compared to Sham-injected stressed rats (Sham-SD vs. uPA-SD; *p* = 0.007), indicating a protective, antidepressant-like effect. However, the administration of doxycycline reversed the uPA effect (uPA-SD vs. Dox-SD; *p* = 0.019), as uPA-overexpressing rats treated with doxycycline displayed latencies similar to those of Sham-injected stressed rats (Sham-SD vs. Dox-SD; *p* = 0.752), demonstrating that doxycycline abrogated the protective effect of uPA. As expected, the statistical analysis showed a significant difference in immobility duration between the groups (H(3) = 20.788, *p* < 0.001). As shown in Figure 6B, multiple comparison tests revealed that Sham-injected stressed rats spent significantly more time immobile compared to single-housed controls (Sham-SH vs. Sham-SD; *p* < 0.001), highlighting increased behavioral despair due to stress exposure. uPA overexpression in the hippocampus without doxycycline significantly reduced the duration of immobility in stressed animals compared to Sham-injected stressed rats (Sham-SD vs. uPA-SD; *p* = 0.012), further suggesting an antidepressant-like effect of uPA. However, in the presence of doxycycline (uPA-SD vs. Dox-SD; *p* = 0.005), the immobility duration in uPA-overexpressing rats was not significantly different from that of Sham-injected stressed rats (Sham-SD vs. Dox-SD; *p* = 0.761), indicating that doxycycline treatment nullified the beneficial effect of uPA overexpression on immobility duration.

#### 3.3.6. Forced Swim Test (FST)

Social defeat has been extensively used to induce behavioral changes in rodents which mimic “depression symptoms”. These symptoms exhibit as behavioral despair, struggling, and immobility. For the latency to the first immobility episode, the statistical analysis did not reveal a significant difference between groups (H(3) = 5.305, *p* = 0.151). Thus, no post hoc analyses were conducted for this parameter, suggesting that social defeat stress and uPA overexpression did not significantly alter the time to initial immobility across the experimental groups (Figure 6C). In contrast, the Kruskal–Wallis test revealed a significant main effect for immobility duration (H(3) = 15.087, *p* = 0.002). As depicted in Figure 6D, post hoc analyses indicated that Sham-injected rats exposed to social defeat stress exhibited significantly longer immobility duration compared to single-housed controls (Sham-SH vs. Sham-SD; *p* = 0.002), indicating heightened behavioral despair due to stress. However, uPA overexpression in the absence of doxycycline significantly reduced immobility duration in stressed animals compared to Sham-injected stressed rats (Sham-SD vs. uPA-SD; *p* = 0.011), suggesting an antidepressant-like effect of uPA. But this effect was reversed by doxycycline (uPA-SD vs. Dox-SD; *p* = 0.017), as uPA-overexpressing rats treated with doxycycline showed immobility durations comparable to those of Sham-injected stressed rats (Sham-SD vs. Dox-SD; *p* = 0.871). Similarly, the same statistical analysis demonstrated a significant effect for immobility episodes/frequency (H(3) = 20.150, *p* < 0.001). As shown in Figure 6E, pairwise comparisons revealed that social defeat stress significantly increased the frequency of immobility episodes in Sham-injected rats compared to single-housed controls (Sham-SH vs. Sham-SD; *p* < 0.001). Hippocampal uPA overexpression without doxycycline significantly reduced the number of immobility episodes in stressed animals (Sham-SD vs. uPA-SD; *p* = 0.014), supporting its antidepressant role. However, in the presence of doxycycline, uPA-overexpressing rats displayed immobility frequencies (uPA-SD vs. Dox-SD; *p* = 0.031) similar to those of Sham-injected stressed rats (Sham-SD vs. Dox-SD; *p* = 0.768), indicating that doxycycline blocked the uPA-mediated reduction in immobility episodes. In addition, the Kruskal–Wallis test revealed a significant effect for climbing episodes/frequency (H(3) = 23.473, *p* < 0.001). As shown in Figure 6F, post hoc analyses indicated that Sham-injected rats exposed to social defeat stress had significantly fewer climbing episodes compared to single-housed controls (Sham-SH vs. Sham-SD; *p* < 0.001), suggesting a reduction in active coping behaviors under stress. However, hippocampal uPA overexpression without doxycycline significantly increased the number of climbing episodes in stressed animals (Sham-SD vs. uPA-SD; *p* = 0.001), indicating a restoration of active coping. In contrast, the presence of doxycycline abrogated this effect (uPA-SD vs. Dox-SD; *p* = 0.011), as uPA-overexpressing rats treated with doxycycline exhibited climbing frequencies similar to Sham-injected stressed rats (Sham-SD vs. Dox-SD; *p* = 0.542), suggesting that doxycycline reversed the uPA-mediated increase in climbing episodes. Finally, the statistical analysis for swimming frequency did not reveal a significant difference between groups (H(3) = 7.184, *p* = 0.055). Therefore, no post hoc analyses were performed for this parameter, indicating that neither social defeat stress nor uPA overexpression significantly influenced swimming behavior across the groups.

### 3.4. uPA Expression Correlated with Anxiety- and Depression-Like Behaviors

**Figure 7:**
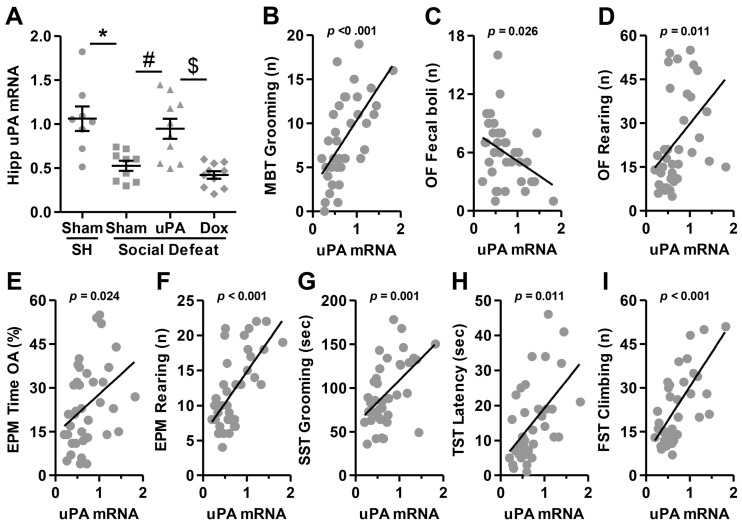
Hippocampal uPA quantification, as measured by RT-PCR and Spearman correlations. (**A**) Data are presented as individual data points with means ± SEM for relative uPA mRNA levels in the hippocampus, as measured by RT-PCR. For the Spearman test, the data represent a simple scatter correlation between uPA mRNA levels, with the (**B**) number of grooming episodes in the MBT, (**C**) number of fecal boli, and (**D**) number of rearing episodes in the OF test, (**E**) the percentage of time spent in the OAs and (**F**) number of rearing episodes in the EPM test, and (**G**) the time spent grooming in the SST, (**H**) latency to the first immobility episode in the TST, and (**I**) number of climbing episodes in the FST. * *p* < 0.05 for Sham-SH vs. Sham-SD; # *p* < 0.05 for Sham-SD vs. uPA-SD; $ *p* < 0.05 for uPA-SD vs. Dox-SD. Sham-SH, n = 8; Sham-SD, n = 9; uPA-SD, n = 10; Dox-SD, n = 10.

After the completion of behavioral testing, brain extraction was performed, and the hippocampus was dissected for uPA mRNA-level assessment across all experimental groups using RT-PCR. Statistical analysis of uPA mRNA expression revealed a significant main effect (H(3) = 17.203, *p* < 0.001), indicating substantial differences among the groups. As illustrated in Figure 7A, post hoc analyses demonstrated that, as anticipated, social defeat stress led to a significant reduction in uPA expression in Sham-injected rats (Sham-SH vs. Sham-SD; *p* = 0.011). However, uPA overexpression significantly restored stress-reduced uPA mRNA levels in socially defeated rats (Sham-SD vs. uPA-SD; *p* = 0.042). Notably, as predicted by the Tet-Off system, doxycycline administration effectively inhibited uPA mRNA overexpression (uPA-SD vs. Dox-SD; *p* = 0.002), bringing uPA expression back to control levels. The presence of doxycycline resulted in uPA expression levels similar to Sham-injected stressed animals (Sham-SD vs. Dox-SD; *p* = 0.306). These findings confirm the regulatory effect of doxycycline on uPA overexpression and its ability to reverse the molecular changes induced by uPA under stress conditions.

To examine the underlying structure of the behavioral data, a principal component analysis (PCA) was conducted. The sampling adequacy was acceptable, with a Kaiser–Meyer–Olkin (KMO) measure of 0.675, indicating that the data were suitable for PCA. Bartlett’s test of sphericity was significant (χ^2^(300) = 693.496, *p* < 0.001), confirming that the correlations between items were sufficiently large for factor analysis. The analysis extracted six components with eigenvalues greater than 1, explaining a total of 73.85% of the variance in the data. The first component had a significantly higher eigenvalue (10.986) and explained 43.94% of the total variance, indicating it accounted for the largest amount of variability in the dataset. The second component contributed 7.74% of the variance, with an eigenvalue of 1.935, bringing the cumulative explained variance to 51.68%. Components three, four, five, and six explained 6.66%, 6.03%, 4.85%, and 4.63% of the variance, respectively, resulting in a cumulative variance of 73.85%. Although each subsequent component accounted for a smaller portion of the total variance, their combined contribution adds valuable dimensional insights into the structure of the data.

The rotated component matrix from the PCA provides valuable insights into the underlying structure of the behavioral variables analyzed across various tests. Table 2 presents the loadings of each behavioral measure on the extracted components, offering a clear understanding of which behaviors cluster together across the six components. Component 1, “stress-induced activity and anxiety-like behaviors”, captures high loadings for several stress-related measures, including TST immobility duration (−0.795), FST climbing duration (0.763), SST grooming duration (0.706), and EPM time in the open arms (0.663). This component appears to reflect a dimension of stress-induced activity or anxiety-like behavior, as seen in the negative loadings of OF fecal boli (−0.637) and SST grooming latency (−0.526). The positive loadings for behaviors such as OF rearing episodes (0.613) and MBT grooming frequency (0.607) further suggest this component represents overall behavioral activity in response to anxiety-inducing environments. Component 2, “latency and hesitancy in exploratory behaviors”, is largely associated with latency and duration measures related to exploratory behaviors, as shown by the high loadings of TST immobility latency (0.811), MBT digging duration (−0.701), and FST immobility duration (−0.695). The positive loading of OF time in the center (0.614) may also indicate that this component encompasses animals’ movement in less protected or more open areas, correlating with hesitancy and latency behaviors. Component 3, “active coping strategies”, loads strongly on FST swimming episodes (0.83), suggesting this component reflects active coping mechanisms in stressful environments. Additionally, positive correlations with SST grooming frequency (0.479) and EPM entries into the open arms (0.555) indicate that this component may also capture aspects of general activity and exploratory behavior in response to stress. Component 4 includes measures such as EPM entries into the open arms (%) (0.7) and OF time in the center (0.303), reflecting behaviors associated with “anxiety and risk-taking”. These variables suggest a relationship between higher exploratory behaviors in anxiety-provoking environments, with further contributions from SST grooming latency (−0.453). Component 5, “behavioral inhibition and immobility frequency”, appears to be characterized by measures related to immobility frequency, as evidenced by loadings on FST immobility frequency (−0.527) and OF line crossing (−0.523). This suggests that this component captures variability in behavioral inhibition or freezing-like behaviors in stressful or unfamiliar environments. Component 6 is highly associated with FST immobility latency (0.901) and may represent animals’ delayed responses to stress-induced immobility, capturing the timing of their immobile states in the face of anxiety-inducing tests.

To enhance the clarity and focus of our findings, we conducted Spearman correlation analyses using standardized Z scores for the behavioral parameters that loaded most strongly onto Component 1, which accounted for the largest proportion of variance (43.94%). Component 1 was closely associated with key stress-related and immobility behaviors, including high loadings from TST immobility duration, FST climbing duration, MBT and SST grooming duration, and EPM time in OAs. These measures are directly relevant to our study’s hypotheses, as they reflect anxiety- and stress-induced behaviors. By utilizing Z scores, we ensured that the behavioral measures were standardized, allowing for more accurate and meaningful correlations with uPA and BDNF expression. Given the centrality of Component 1 to our research questions, these correlations are presented below, as they provide the most direct insight into the relationship between behavioral responses and molecular markers. In contrast, the behavioral parameters associated with Components 2–6 explained smaller proportions of variance and were more reflective of exploratory behaviors, latency to action, and risk taking. While these components offer valuable information about broader behavioral constructs, their analyses are included in the supplementary materials to maintain a focused presentation of the most significant findings in the main text. Correlations using Z scores for these components are provided in Supplementary Table S1, ensuring comprehensive coverage of all relevant behavioral dimensions.

Spearman correlation analyses were conducted to assess the relationship between hippocampal uPA mRNA expression and behavioral measures of anxiety and depression. In the MBT, a significant positive correlation was found between uPA mRNA and grooming frequency (*r*_(37)_ = 0.616, *p* < 0.001; Figure 7B), suggesting an association between uPA overexpression and increased grooming behavior. In the OF test, hippocampal uPA mRNA expression was significantly negatively correlated with the number of fecal boli (*r*_(37)_ = −0.365, *p* = 0.026; Figure 7C), while a positive correlation was observed between uPA mRNA and the number of rearing episodes (*r*_(37)_ = 0.415, *p* = 0.011; Figure 7D), indicating that higher uPA levels were linked to reduced anxiety-like behaviors. In the EPM, uPA mRNA was positively correlated with both the time spent in the OAs (*r*_(37)_ = 0.371, *p* = 0.024; Figure 7E) and the number of rearing episodes (*r*_(37)_ = 0.642, *p* < 0.001; Figure 7F), further supporting the anxiolytic role of uPA overexpression. In the SST, uPA mRNA was significantly positively correlated with total grooming time (*r*_(37)_ = 0.513, *p* = 0.001; Figure 7G), while in the TST, uPA mRNA was negatively correlated with immobility duration (*r*_(37)_ = −0.416, *p* = 0.011; Figure 7H), indicating an antidepressant effect. Lastly, in the FST, a strong positive correlation was found between uPA mRNA expression and climbing behavior (*r*_(37)_ = 0.642, *p* < 0.001; Figure 7I), further indicating that uPA overexpression mitigated depressive-like behaviors.

Detailed correlations between uPA mRNA expression and additional behavioral measures from Components 2–6 are presented in Supplementary Table S1. These correlations, while not the primary focus of the main text, provide valuable insights into the broader range of behaviors assessed in this study, offering a comprehensive understanding of the influence of uPA overexpression on various behavioral domains.

### 3.5. BDNF Expression Correlated with Anxiety- and Depression-Like Behaviors

**Figure 8:**
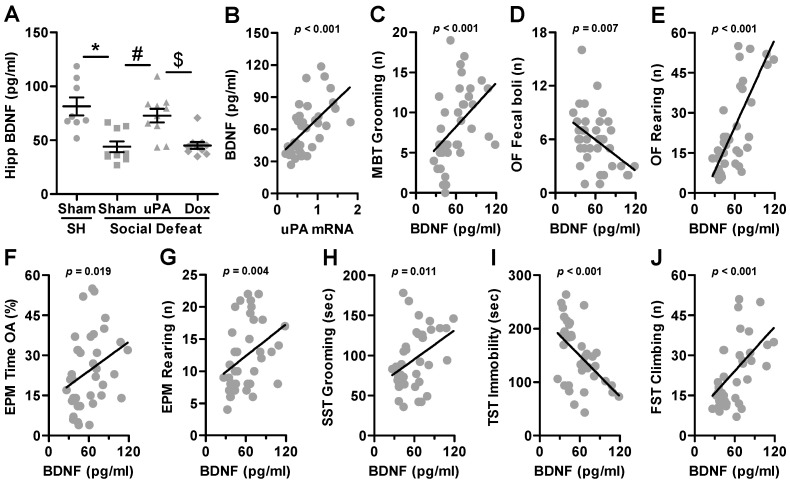
Hippocampal BDNF quantification, as measured by ELISA and Spearman correlations. (**A**) Data are presented as individual data points with means ± SEM for BDNF proteins levels in the hippocampus, as measured by ELISA. For the Spearman test, the data represent a simple scatter correlation between BDNF levels, with (**B**) the hippocampal uPA mRNA expression, (**C**) number of grooming episodes in the MBT, (**D**) number of fecal boli, and (**E**) number of rearing episodes in the OF test, (**F**) the percentage of time spent in the OAs and (**G**) number of rearing episodes in the EPM test, and (**H**) the time spent grooming in the SST, (**I**) latency to the first immobility episode in the TST, and (**J**) number of climbing episodes in the FST. * *p* < 0.05 for Sham-SH vs. Sham-SD; # *p* < 0.05 for Sham-SD vs. uPA-SD; $ *p* < 0.05 for uPA-SD vs. Dox-SD. Sham-SH, n = 8; Sham-SD, n = 9; uPA-SD, n = 10; Dox-SD, n = 10.

Given the role of hippocampal uPA in reversing social defeat stress-elicited behaviors related to mood disorders, we sought to investigate the underlying mechanism. Previous studies suggest that uPA is involved in pro-BDNF cleavage and BDNF maturation [79]. Therefore, BDNF protein levels were quantified in hippocampal brain samples using ELISA test as described in the Methods section, and the results are shown in Figure 8. The Kruskal–Wallis test revealed a statistically significant difference across experimental groups (H(3) = 21.649; *p* < 0.001). As depicted in Figure 8A, post hoc pairwise comparisons revealed that socially defeated rats injected with Sham vectors exhibited a significant reduction in BDNF levels compared to controls (Sham-SH vs. Sham-SD, *p* < 0.001). However, BDNF levels significantly increased following uPA overexpression, reversing the effects of stress (Sham-SD vs. uPA-SD, *p* < 0.001). Crucially, in uPA-injected rats, the administration of doxycycline inhibited this effect, leading to a reduction in hippocampal BDNF levels (uPA-SD vs. Dox-SD, *p* = 0.003), restoring levels to that of control rats (Sham-SD vs. Dox-SD, *p* = 0.761). This suggests that the uPA-mediated regulation of BDNF may underlie its protective effects against social stress-induced mood disturbances.

Notably, a strong positive correlation was found between uPA expression and BDNF levels in the hippocampus (*r*_(37)_ = 0.624, *p* < 0.001; Figure 8B). Next, we sought to examine the correlation between BDNF levels and the behavioral parameters that loaded most strongly onto Component 1, as described above. In the MBT, the correlation coefficient for the difference between the BDNF expression and grooming frequency was positive and the two parameters correlated significantly (*r*_(37)_ = 0.573, *p* < 0.001; Figure 8C). In the OF test, the Spearman analysis revealed a strong negative correlation between hippocampal BDNF expression and the number of fecal boli (*r*_(37)_ = −0.434, *p* = 0.007; Figure 8D). However, a strong positive correlation was found between BDNF expression and the number of rearing episodes (*r*_(37)_ = 0.710, *p* < 0.001; Figure 8E). In the EPM, a significant positive correlation was found between BDNF and the time spent in the OAs (*r*_(37)_ = 0.385, *p* = 0.019; Figure 8F), as well as with the number of rearing episodes (*r*_(37)_ = 0.459, *p* = 0.004; Figure 8G). In the SST, the correlation between BDNF expression and the total time spent grooming was positive (*r*_(37)_ = 0.411, *p* = 0.011; Figure 8H). In the TST, a strong negative correlation was found between BDNF protein expression and immobility duration (*r*_(37)_ = −0.520, *p* < 0.001; Figure 8I). Finally, in the FST, a strong positive correlation was found between BDNF levels and climbing behavior (*r*_(37)_ = 0.532, *p* < 0.001; Figure 8J). The Spearman correlation analyses between BDNF expression and the behavioral parameters identified across the various components are presented in Supplementary Table S1. These correlations provide additional insights into the association between BDNF levels and key behavioral measures, complementing the primary findings related to uPA expression.

It is important to note that uPA mRNA and BDNF protein levels were also isolated from the dorsal striatum (DS), quantified, and analyzed for potential Spearman correlations with anxiety- and depression-like behavioral parameters. However, the results revealed no significant correlations between uPA mRNA or BDNF protein levels in the DS and any of the behavioral measures assessed (Supplementary Table S2).

## 4. Discussion

To the best of our knowledge, this is the first study to assess the role of local hippocampal uPA manipulation in stress-related mood disorders. The key findings of the present study were first, exposure to social defeat stress altered uPA expression in the hippocampus, a key brain region associated with stress-induced anxiety- and depression-like behaviors. Second, lentiviral uPA gain-of-function in the hippocampus abrogated social defeat stress-elicited depressant and anxiogenic-like effects. Finally, ectopic uPA overexpression protective effects were accompanied by increased hippocampal BDNF protein levels. Taken together, these data strongly suggest that, at least in the hippocampus, uPA is a key player in stress-elicited mood disorders, most probably via a pro-BDNF maturation-related mechanism.

In our first experiment, we found a significant decrease in uPA expression in socially defeated rats compared to their single-housed controls. This alteration was mainly observed in the hippocampus and in the nucleus accumbens, but not in the dorsal striatum. Several important members of the fibrinolytic system, such as uPA, are linked to synaptic plasticity and seem to be altered both in the periphery and in the CNS following stress exposure. For example, high levels of soluble serum uPA receptor were associated with an increased risk of previous and subsequent use of antidepressant medications in both healthy men and women [80]. Also, serum uPA receptor levels were higher in major depressive disorder (MDD) and in patients who had recently attempted suicide [81]. In addition, the signaling cascade implicated in stress-induced uPA expression and/or activation is most probably regulated by proinflammatory cytokines [82], indicating that environmental and/or social stressors may regulate uPA activity in the brain. Taken together, we hypothesize that altered uPA expression in the hippocampus may be involved in chronic social stress-related mood disorders such as anxiety and depression.

We found that a 10-day stress exposure significantly altered animals’ physiology. In fact, socially defeated rats did not gain as much weight as their control counterparts, although they consumed comparable amounts of food. These data confirm previously published studies in which chronic social stress resulted in significant bodyweight loss, which is considered one of the most marked and consistent consequences of subordination stress in both rats and mice in different social stress models [83,84,85]. One plausible explanation for bodyweight alteration observed in subordinate animals might be the loss of both adipose and lean tissue, with preferential retention of visceral adiposity, as reported by Tamashiro and colleagues [86,87]. Furthermore, socially defeated rats consumed more water than single-housed controls, suggesting increased stress-induced dehydration, which agrees with previous findings in both rats and mice [88,89]. We hypothesize that the increased fluid intake during social defeat stress was mainly due to dehydration. Nevertheless, modifications in the vasopressin and/or angiotensin systems, as well as changes to the hypothalamus, may, at least in part, be responsible for the shift in drinking behavior [90]. It is worth mentioning that thirst and increased water intake are associated with stress exposure [90,91]. Taken together, these findings support the utility of this model to evaluate stress-related mood disorders by revealing high stress susceptibility for the socially defeated rats.

To further explore the behavioral significance of decreased hippocampal uPA levels for chronic social stress, we assessed the effect of uPA manipulation on stress-induced anxiety- and depression-like behaviors in adult rats. We found that defeat stress of 10 consecutive days induced substantial alterations in anxiety- and depression-like behaviors, which is in line with the current literature [92]. Using the MBT, we found that ectopic uPA expression was efficient in reducing social defeat-exacerbated digging and marble-burying behaviors in rats. This indicates a disruption of the repetitive/compulsive behavior phenotype associated with social stress exposure that was normalized following hippocampal uPA overexpression. Moreover, social defeat stress increased the grooming latency and decreased the total time of grooming behavior in the sucrose splash test, suggesting lower self-care-motivated drive [93]. Collectively, these findings indicate that disruption of the self-care behavior phenotype associated with stress exposure was abrogated following hippocampal uPA overexpression. In the EPM and OF tests, socially defeated rats displayed increased anxiety-like behavior, as demonstrated by the reduced time spent in the center of the arena, increased number of fecal boli, and decreased time and number of entries into the open arms of the maze, indicative of the anxiogenic-like effect of stress exposure. Therefore, uPA overexpression did not appear to affect spontaneous motor activity but increased the time and entries into the OAs, as well as rearing behavior, suggesting that, following social defeat stress, hippocampal uPA gain-of-function elicited an anxiolytic-like response in the OF and EPM tests. Interestingly, uPA gain-of-function prevented a social defeat-induced increase in immobility in the TST and FST, suggesting that hippocampal uPA overexpression could reverse social defeat stress-evoked depression-like behavior in the TST and FST. The absence of locomotor changes during the performance of the EPM and OF tests suggests that social defeat-exposed rats have normal motor function; hence, excluding presumed motor deficits may be responsible for the observed uPA antidepressant-like properties. Taken together, according to the conventional interpretation of these behaviors, hippocampal uPA overexpression appears to be a promising antidepressant and anxiolytic player in laboratory animals.

Viral-mediated uPA overexpression in the hippocampus prevented stress-induced anxiety-like behavior in both the EPM and OF tests. In other words, our findings proved that, in male rats, uPA gain-of-function has a potential to act as an effective anxiolytic. Accordingly, and compared to their wild-type littermates, uPA transgenic mice showed decreased exploratory activity [18]. Although uPA knockout mice were reported to display a non-anxious phenotype, Rantala and colleagues concluded that uPA ablation was associated with exacerbated emotional reactivity to unanticipated aversive stimuli and reduced curiosity about environmental surroundings [18]. It is unknown why there appears to be no agreement between our study and the one conducted by Rantala and co-workers. Nevertheless, it is possible to hypothesize that these differences might, at least in part, be explained by unexplored functional compensating mechanisms that are typical of conventional knockout mice. Additionally, developmental neuronal changes, which are frequently observed in transgenic mice, may possibly be the cause of the seemingly contradictory data. The result of this study should be interpreted within its limitations; only males were used, while females are more prone to develop depression- and anxiety-like behaviors overall. This is a caveat to this study, and the current findings should be interpreted with caution. It is also important to emphasize the exploratory nature of our study; the current findings provide a strong basis for the use of lentiviral vector-mediated gene transfer technologies to locally alter gene expression and protease levels in rodents’ brain tissue.

In our study, we conducted Spearman correlation analyses to explore the potential relationship between uPA mRNA expression levels and the parameters of anxiety- and depression-like behavior. We found that hippocampal uPA mRNA expression showed a significant negative correlation with measures of anxiety-like behavior, such as the time spent in the open arms of the EPM and the number of entries into the open arms. These findings suggest that higher levels of hippocampal uPA mRNA expression may be associated with reduced anxiety-like behavior in our experimental model. Additionally, we observed a significant positive correlation between hippocampal uPA mRNA expression and parameters of depression-like behavior, as indicated by increased immobility time in the FST and TST.

The results of the PCA offer important insights into the behavioral dimensions underlying responses to stress and anxiety-like behaviors, and how they may relate to the overexpression of uPA in the hippocampus. The PCA extracted six components, with Component 1 explaining the largest proportion of the variance (43.94%) and representing behaviors central to the study’s investigation of stress-induced immobility and activity. The correlation of these behavioral patterns with uPA and BDNF expression provides a comprehensive framework to understand the potential anxiolytic and antidepressant role of uPA overexpression.

Component 1, “stress-induced activity and anxiety-like behaviors”, which includes measures such as TST immobility duration, FST climbing duration, SST grooming duration, and EPM time in the open arms, clearly reflects behaviors associated with stress and anxiety. High loadings on immobility measures suggest that this component captures a significant dimension of behavioral inhibition and stress responses, key markers of depressive-like behavior in rodents. The negative loadings on immobility and the positive loadings on active behaviors (such as grooming and climbing) imply that animals exhibiting higher activity levels under stress are better able to cope with anxiety-inducing environments. Given that uPA has been shown to play a role in neural plasticity and neuroprotection, its overexpression in the hippocampus may mitigate the depressive-like behaviors associated with prolonged immobility. The hippocampus is integral to the regulation of stress responses, and the increase in uPA could facilitate synaptic remodeling or protect against stress-induced neurodegeneration, potentially reversing immobility behaviors linked to depressive states. The significant role of uPA in enhancing neuroplasticity might thus encourage more active, exploratory behaviors under stressful conditions, reflected in the positive loadings of climbing and grooming behaviors in Component 1.

The high loadings for anxiety-like behaviors, such as EPM time in the open arms and OF rearing episodes, further support the idea that Component 1 (“Stress-Induced Activity and Anxiety-Like Behaviors”) encapsulates an important anxiety-related dimension. Increased time in the open arms of the EPM suggests reduced anxiety, which could be linked to the anxiolytic effects of uPA overexpression. By enhancing synaptic plasticity and neurogenesis, particularly in stress-sensitive regions like the hippocampus, uPA may reduce anxiety-like behaviors, as observed in animals that spend more time exploring less protected environments (the open arms of the EPM). These findings align with previous studies showing that the overexpression of uPA can lead to increased hippocampal resilience to stress and enhanced coping behaviors in anxiety-provoking contexts. The behavioral data suggest that uPA overexpression allows animals to exhibit less immobility and more exploratory activity in response to social stress, potentially through the modulation of hippocampal BDNF signaling pathways. BDNF has been widely implicated in stress resilience and the alleviation of depressive symptoms, and its interaction with uPA could further enhance synaptic connectivity, reducing stress-induced behavioral impairments.

Component 2, “latency and hesitancy in exploratory behaviors”, captures latency measures, such as TST immobility latency and MBT digging duration, which may reflect animals’ hesitancy or delay in responding to stress. This component could highlight a behavioral dimension where uPA overexpression influences not just the total amount of immobility, but also the speed at which animals engage in active coping mechanisms after stress exposure. Prolonged latencies may indicate heightened anxiety or impaired coping ability, which could be mitigated by uPA’s influence on synaptic function. Component 3, “active coping strategies”, with high loadings on FST swimming episodes and SST grooming frequency, represents a distinct behavioral construct associated with active coping strategies, such as swimming in the FST. This component suggests that animals exhibiting increased uPA levels may show more active responses to stress, as reflected by higher swimming activity, which is often interpreted as a marker of antidepressant-like behavior. Component 5, “behavioral inhibition and immobility frequency”, is characterized by immobility frequency and lower activity, with variables like FST immobility frequency and OF line crossing loading negatively. This may reflect a distinct behavioral dimension where uPA overexpression could reduce the frequency of immobility episodes, particularly in tests like the FST and OF, where stress-related immobility is a hallmark of depressive-like behavior. A reduction in immobility frequency would further support the hypothesis that uPA overexpression confers resilience to stress-induced behavioral suppression.

The PCA findings, particularly the centrality of Component 1, “stress-induced activity and anxiety-like behaviors”, in explaining behavioral variance, suggest that the anxiolytic and antidepressant effects of uPA overexpression manifest primarily through reducing immobility and promoting active stress-coping behaviors. The contributions of Component 2, “latency and hesitancy in exploratory behaviors”, and Component 3, “active coping strategies”, particularly in the latency and exploratory dimensions, further support the multifaceted role of uPA in mitigating the effects of chronic social stress exposure. By understanding how uPA modulates these behavioral constructs, particularly through its interactions with BDNF, we can better appreciate its potential therapeutic role in disorders characterized by chronic stress, anxiety, and depression.

These findings are consistent with the hypothesis that uPA’s neuroplastic and neuroprotective effects enhance hippocampal function, leading to improved behavioral outcomes in stress-challenged animals. Taken together, the PCA results underscore the complexity of stress- and anxiety-related behaviors and how uPA overexpression in the hippocampus may influence various aspects of these responses. Component 1, “stress-induced activity and anxiety-like behaviors”, in particular, highlights the key role of uPA in reducing immobility and enhancing active coping behaviors, while other components reveal additional dimensions related to latency, exploration, and immobility frequency. Together, these findings provide a comprehensive behavioral framework through which the molecular effects of uPA overexpression may be understood, offering valuable insights into its therapeutic potential for alleviating the effects of social stress on anxiety and depression.

Presently, the protective mechanisms of hippocampal uPA gain-of-function are still unknown. However, we hypothesize that BDNF might play a central role in these behavioral alterations. In fact, to assess the role of uPA overexpression, we measured the levels of BDNF protein expression. The results show that increased BDNF was correlated with elevated uPA levels in the hippocampus. In our investigation, we employed Spearman correlation analyses to explore potential associations between hippocampal BDNF expression, as measured by ELISA, and parameters indicative of anxiety and depression-like behaviors. Our findings revealed intriguing relationships between hippocampal BDNF levels and behavioral phenotypes. Specifically, we observed a significant positive correlation between hippocampal BDNF expression and indicators of reduced anxiety-like behavior. These results suggest that higher levels of hippocampal BDNF expression may be linked to decreased anxiety-like behaviors in our experimental model. Conversely, we also observed a significant negative correlation between hippocampal BDNF expression and parameters indicative of depression-like behavior. This suggests that higher levels of hippocampal BDNF expression may be associated with diminished depression-like behaviors. Taken together, our findings suggest a potential role for hippocampal BDNF expression in modulating both anxiety and depression-like behaviors, underscoring the intricate involvement of BDNF in the neurobiology of mood regulation. Further research is warranted to elucidate the underlying molecular mechanisms and to explore the therapeutic implications of these findings in the context of mood disorders.

Although the specific mechanisms of how uPA is implicated in neuronal response modulation are not fully understood, we hypothesize that it is involved in pro-BDNF maturation through plasmin activation. Consistent with this hypothesis, we found that uPA’s expression in the hippocampus is altered following stress exposure. Also, and in support of our hypothesis, Karagyaur and colleagues reported that non-viral plasmid constructions containing uPA and BDNF genes restored nerves’ structure and function and stimulated nerve regeneration following traumatic injury in mice [94]. Therefore, we speculate that mature BDNF generation downstream of the plasmin system [95] may contribute to the uPA anxiolytic- and antidepressant-like response observed following stress exposure. There is a growing body of evidence indicating that social stress impairs BDNF expression in limbic brain regions involved in mood control. In fact, reduced hippocampal BDNF levels were observed in patients suffering from depression [96], and increased BDNF levels were observed following antidepressant treatments [97]. Likewise, in rodents, chronic unpredictable mild stress was shown to decrease BDNF levels in the hippocampus [98]. We were able to show in a previous study that lentiviral-mediated overexpression of BDNF in the hippocampus elicited an antidepressant- and anxiolytic-like response in rats subjected to seven days of social defeat [26]. The latter observation was in line with a previous report showing that bilateral infusion of recombinant BDNF into the dentate gyrus of the hippocampus elicited an antidepressant-like behavior, as it increases the swimming behavior of male Sprague Dawley rats [99]. Also, BDNF-overexpressing transgenic mice, including in the hippocampus, exhibited an improved performance in the FST [100]. However, heterozygous BDNF mice, subjected to CUMS, exhibited a depressive-like response in the FST [101], with these same mice not being responsive to antidepressants in the FST [102]. Moreover, moderate acute stress triggers the rapid release of catecholamines and activation of the plasmin system, which in turn increases BDNF levels and enhances synaptic transmission, leading to improved learning and decision-making abilities [103]. Severe or chronic stress triggers the release of catecholamines, which might be expected to enhance decision-making, working memory, and mood. However, elevated cortisol levels associated with distress lead to the increased production of PAI-1, inhibiting the cleavage of pro-BDNF by plasmin and disrupting BDNF maturation. Consequently, chronic stress leads to neuronal apoptosis and alterations in connectivity in the hippocampus and prefrontal cortex [103], impairing working memory, decision-making processes, as well as mood disorders. In fact, emerging evidence suggests that MDD might be related to elevated PAI-1 levels in both humans and animals [10,79,104,105]. Taken together, these findings suggest that intact BDNF signaling is required for antidepressant-improved behavioral effects following stress exposure.

## 5. Limitations and Future Directions

There are a few caveats to this study and, accordingly, the current findings should be interpreted with caution. Firstly, our study employed a sample size of 8–10. While this allowed us to explore interesting trends and gather valuable data, it is important to acknowledge the limitations associated with a smaller sample size. In fact, smaller samples may not be fully representative, and the results might not be generalized. Also, smaller sample sizes can lead to lower statistical power. This means the study might be less likely to detect true effects, potentially resulting in false negative conclusions (failing to reject the null hypothesis when it is actually false). Due to the sample size limitations, our findings should be considered exploratory in nature. They provide a valuable starting point for further investigation but do not offer definitive conclusions, and replication of these findings with larger, more representative samples is necessary to confirm their validity and generalizability. Nevertheless, our results can be used to generate specific hypotheses for future studies with larger sample sizes and more robust statistical analyses. Secondly, one limitation of correlating BDNF expression using ELISA with uPA mRNA lies in the indirect nature of this association. While changes in uPA mRNA expression may suggest alterations in the transcriptional regulation of the uPA gene, it does not directly confirm changes in uPA protein levels or activity. This discrepancy between mRNA and protein levels can arise from various factors, including post-transcriptional modifications, translational regulation, and protein degradation processes. Therefore, relying solely on uPA mRNA levels to infer the impact of uPA on BDNF expression may overlook important nuances in protein dynamics. Consequently, interpreting the role of uPA in stress-induced depression-like behavior based solely on mRNA expression data may be limited, as they do not provide a direct measure of uPA activity or its downstream effects on BDNF signaling pathways. Addressing this limitation may require additional experiments, such as protein-level analyses or functional assays, to validate the relationship between uPA and BDNF and better understand their contributions to stress-related neurobiology and depressive behaviors. Thirdly, the study comprised four experimental groups: (1) Sham + no chronic stress, (2) Sham + chronic stress, (3) uPA + chronic stress, and (4) uPA + doxycycline + chronic stress. The inclusion of an additional group, uPA + no chronic stress, would have been highly beneficial. With only these four groups, it remains unclear to what extent uPA overexpression influenced control animals. In nearly all comparisons, there is no statistically significant distinction between group 1 (Sham-SH) and group 3 (uPA-SD). However, this fails to clearly elucidate the impact of uPA on the variables measured. This ambiguity arises because it is uncertain how animals would have responded to uPA overexpression without chronic stress. Finally, female rats should have been included in this study. Previous findings have shown significant differences in stress susceptibility and resilience between males and females [106,107,108,109,110]. Also, depression is more prevalent in women than in men. However, it is acknowledged that there are challenges in employing an appropriate chronic social defeat stress paradigm in females. Interestingly, there are emerging studies reporting a new version of the social defeat paradigm that is effective in female mice [111,112,113,114,115]. Although this study provides valuable insights, a notable limitation is the exclusive use of male rats, potentially overlooking sex-specific responses that may occur concerning the effects of uPA overexpression on social stress resilience. Future research endeavors are poised to tackle this issue by incorporating female rats into the study design, leading to a deeper understanding of the use of plasmin system activation to mitigate the adverse effects of social stress.

## 6. Conclusions

Our data suggest that overexpression and/or activation of uPA prevents chronic social stress-deteriorated emotions partially by activating BDNF maturation. Considering the significant expression of uPA in mood-associated brain regions, these findings indicate a potential mechanism for chronic social defeat-elicited behavioral alterations via BDNF modulation. Although more research is needed to understand mechanistic links between uPA activation, BDNF maturation, and emotion diseases, our study may open new therapeutic avenues focused on tackling stress-induced mood disorders.

## Figures and Tables

**Table 1:** Experimental timeline. Timeline showing the sequence of events and behavioral testing the animals underwent.

Days	Procedure
1–10	Social defeat (food and water intake)
11	Stereotaxic surgery/viral injection
21	Marble burying test (MBT)
22	Sucrose splash test (SST)
23	Open field (OF) test
24	Elevated plus maze (EPM) test
25	Tail suspension test (TST)
26	Forced swim test (FST)
27	Animals’ sacrifice

**Table 2:** Component loadings from the principal component analysis (PCA) of behavioral measures.

	Components					
	1	2	3	4	5	6
TST immobility duration	−0.795	−0.308				0.166
FST climbing duration	0.763					
SST grooming duration	0.706			0.458		
EPM time in OAs (%)	0.663			0.381		
OF fecal boli	−0.637					−0.321
OF rearing episodes	0.613		0.406			
MBT grooming episodes	0.607					0.522
SST grooming latency	−0.526		−0.453		−0.384	
EPM rearing frequency	0.435		0.415			
TST immobility latency		0.811				0.304
MBT digging duration	−0.493	−0.701				
FST immobility duration	−0.333	−0.695				
MBT buried marbles	−0.429	−0.689			−0.328	
OF time in center		0.614	0.303			
MBT digging latency	0.497	0.524				
FST swimming episodes			0.83			
EPM entries into OAs (n)		0.424	0.555	0.524		
SST rearing frequency	0.361		0.548			0.498
SST grooming frequency		0.479	0.497			0.329
EPM entries into CAs (n)				−0.784		
EPM entries into OAs (%)		0.338	0.446	0.7		
EPM grooming frequency					0.772	
FST immobility frequency	−0.401	−0.32			−0.527	
OF line crossing		0.448		0.366	−0.523	
FST immobility latency						0.901

## Data Availability

Data available on reasonable request.

## Supplementary Materials

The following supporting information can be downloaded at https://www.mdpi.com/article/10.3390/biom14121603/s1, Table S1: Correlations between hippocampal uPA mRNA (upper part) and BDNF protein levels (lower part) and measures of anxiety- and depression-like behaviors. Table S2: Correlations between dorsal striatum uPA mRNA (upper part) and BDNF protein levels (lower part) and measures of anxiety- and depression-like behaviors.
