# Supplementary material for: Hippocampal Viral-Mediated Urokinase Plasminogen Activator (uPA) Overexpression Mitigates Stress-Induced Anxiety and Depression in Rats by Increasing Brain-Derived Neurotrophic Factor (BDNF) Levels
Source: Biomolecules. 2024 Dec 15;14(12):1603. doi: 10.3390/biom14121603 (PMC11674468; doi:10.3390/biom14121603)
Supplement: Supplementary file 1 [file biomolecules-14-01603-s001.zip › biomolecules-3208926-supplementary.pdf]

	Marble burying test (MBT)	Open field (OF) test	Elevated Plus maze (EPM) test	Sucrose splash test (SST)	Tail suspension test (TST)	Forced swim test (FST)
**Hippocampal uPA mRNA**	**Digging latency (sec)**<br>$r_{(37)} = 0.371$<br>$p = 0.024$<br>**Digging time (sec)**<br>$r_{(37)} = -0.522$<br>$p < 0.001$<br>**Buried marbles (n)**<br>$r_{(37)} = -0.473$<br>$p = 0.003$	**Time in center (sec)**<br>$r_{(37)} = 0.305$<br>$p = 0.067$<br>**Line crossing (n)**<br>$r_{(37)} = -0.097$<br>$p = 0.567$<br>**Rearing frequency (n)**<br>$r_{(37)} = 0.495$<br>$p = 0.002$	**Entries in OA (n)**<br>$r_{(37)} = 0.486$<br>$p = 0.002$<br>**Entries in OA (%)**<br>$r_{(37)} = 0.421$<br>$p = 0.009$<br>**Entries in CA (n)**<br>$r_{(37)} = -0.060$<br>$p = 0.726$<br>**Grooming frequency (n)**<br>$r_{(37)} = 0.282$<br>$p = 0.091$	**Grooming latency (sec)**<br>$r_{(37)} = -0.612$<br>$p < 0.001$<br>**Grooming frequency (n)**<br>$r_{(37)} = 0.607$<br>$p < 0.001$<br>**Rearing frequency (n)**<br>$r_{(37)} = 0.497$<br>$p = 0.002$	**Immobility latency(sec)**<br>$r_{(37)} = 0.534$<br>$p < 0.001$	**Immobility latency (sec)**<br>$r_{(37)} = 0.652$<br>$p < 0.001$<br>**Immobility time (sec)**<br>$r_{(37)} = -0.409$<br>$p = 0.012$<br>**Immobility frequency (n)**<br>$r_{(37)} = -0.485$<br>$p = 0.002$<br>**Swimming frequency (n)**<br>$r_{(37)} = 0.265$<br>$p = 0.112$
**Hippocampal BDNF proteins**	**Digging latency (sec)**<br>$r_{(37)} = 0.524$<br>$p < 0.001$<br>**Digging time (sec)**<br>$r_{(37)} = -0.617$<br>$p < 0.001$<br>**Buried marbles (n)**<br>$r_{(37)} = -0.666$<br>$p < 0.001$	**Time in center (sec)**<br>$r_{(37)} = 0.575$<br>$p < 0.001$<br>**Line crossing (n)**<br>$r_{(37)} = -0.229$<br>$p = 0.173$<br>**Rearing frequency (n)**<br>$r_{(37)} = 0.710$<br>$p < 0.001$	**Entries in OA (n)**<br>$r_{(37)} = 0.458$<br>$p = 0.004$<br>**Entries in OA (%)**<br>$r_{(37)} = 0.356$<br>$p = 0.31$<br>**Entries in CA (n)**<br>$r_{(37)} = 0.176$<br>$p = 0.297$<br>**Grooming frequency (n)**<br>$r_{(37)} = 0.412$<br>$p = 0.011$	**Grooming latency (sec)**<br>$r_{(37)} = -0.662$<br>$p < 0.001$<br>**Grooming frequency (n)**<br>$r_{(37)} = 0.588$<br>$p < 0.001$<br>**Rearing frequency (n)**<br>$r_{(37)} = 0.523$<br>$p < 0.001$	**Immobility latency(sec)**<br>$r_{(37)} = 0.542$<br>$p < 0.001$	**Immobility latency (sec)**<br>$r_{(37)} = 0.364$<br>$p = 0.027$<br>**Immobility time (sec)**<br>$r_{(37)} = -0.483$<br>$p = 0.002$<br>**Immobility frequency (n)**<br>$r_{(37)} = -0.519$<br>$p = 0.001$<br>**Swimming frequency (n)**<br>$r_{(37)} = 0.233$<br>$p = 0.166$

Suppl. Table S1 Correlations between hippocampal uPA mRNA (upper part) and BDNF protein levels (lower part) and measures of anxiety- and depression-like behaviors.

	Marble burying test (MBT)	Open field test (OF)	Elevated Plus maze test (EPM)	Sucrose splash test (SST)	Tail suspension test (TST)	Forced swim test (FST)
Dorsal striatum uPA mRNA	**Digging latency (sec)**<br>$r_{(37)}$ = -0.042<br>p = 0.805	**Time in center (sec)**<br>$r_{(37)}$ = 0.34<br>p = 0.841	**Time in OA (%)**<br>$r_{(37)}$ = -0.048<br>p = 0.777	**Grooming latency (sec)**<br>$r_{(37)}$ = 0.053<br>p = 0.755	**Immobility latency (sec)**<br>$r_{(37)}$ = -0.031<br>p = 0.857	**Immobility latency (sec)**<br>$r_{(37)}$ = 0.027<br>p = 0.874
	**Digging time (sec)**<br>$r_{(37)}$ = 0.106<br>p = 0.534	**Fecal boli (n)**<br>$r_{(37)}$ = 0.066<br>p = 0.699	**Entries in OA (n)**<br>$r_{(37)}$ = 0.004<br>p = 0.983	**Grooming time (sec)**<br>$r_{(37)}$ = -0.032<br>p = 0.849	**Immobility time (sec)**<br>$r_{(37)}$ = 0.094<br>p = 0.580	**Immobility time (sec)**<br>$r_{(37)}$ = 0.102<br>p = 0.547
	**Buried marbles (n)**<br>$r_{(37)}$ = -0.074<br>p = 0.662	**Rearing frequency (n)**<br>$r_{(37)}$ = -0.106<br>p = 0.531	**Entries in OA (%)**<br>$r_{(37)}$ = 0.061<br>p = 0.721	**Grooming frequency (n)**<br>$r_{(37)}$ = -0.067<br>p = 0.692		**Immobility frequency (n)**<br>$r_{(37)}$ = -0.021<br>p = 0.907
	**Grooming time (sec)**<br>$r_{(37)}$ = -0.007<br>p = 0.966	**Line cross (n)**<br>$r_{(37)}$ = 0.201<br>p = 0.234	**Entries in CA (n)**<br>$r_{(37)}$ = -0.199<br>p = 0.237	**Rearing frequency (n)**<br>$r_{(37)}$ = -0.129<br>p = 0.447		**Immobility frequency (n)**<br>$r_{(37)}$ = -0.183<br>p = 0.279
			**Grooming frequency (n)**<br>$r_{(37)}$ = -0.172<br>p = 0.310			**Climbing frequency (n)**<br>$r_{(37)}$ = -0.004<br>p = 0.982
Dorsal striatum BDNF proteins	**Digging latency (sec)**<br>$r_{(37)}$ = -0.009<br>p = 0.956	**Time in center (sec)**<br>$r_{(37)}$ = 0.122<br>p = 0.473	**Time in OA (%)**<br>$r_{(37)}$ = 0.031<br>p = 0.853	**Grooming latency (sec)**<br>$r_{(37)}$ = -0.098<br>p = 0.563	**Immobility latency (sec)**<br>$r_{(37)}$ = 0.258<br>p = 0.124	**Immobility latency (sec)**<br>$r_{(37)}$ = 0.160<br>p = 0.345
	**Digging time (sec)**<br>$r_{(37)}$ = -0.253<br>p = 0.131	**Fecal boli (n)**<br>$r_{(37)}$ = -0.227<br>p = 0.177	**Entries in OA (n)**<br>$r_{(37)}$ = 0.040<br>p = 0.815	**Grooming time (sec)**<br>$r_{(37)}$ = -0.072<br>p = 0.673	**Immobility time (sec)**<br>$r_{(37)}$ = -0.041<br>p = 0.810	**Immobility time (sec)**<br>$r_{(37)}$ = -0.230<br>p = 0.172
	**Buried marbles (n)**<br>$r_{(37)}$ = -0.122<br>p = 0.471	**Rearing frequency (n)**<br>$r_{(37)}$ = 0.027<br>p = 0.874	**Entries in OA (%)**<br>$r_{(37)}$ = 0.062<br>p = 0.716	**Grooming frequency (n)**<br>$r_{(37)}$ = 0.169<br>p = 0.317		**Immobility frequency (n)**<br>$r_{(37)}$ = -0.019<br>p = 0.912
	**Grooming time (sec)**<br>$r_{(37)}$ = 0.132<br>p = 0.437		**Entries in CA (n)**<br>$r_{(37)}$ = 0.027<br>p = 0.872	**Rearing frequency (n)**<br>$r_{(37)}$ = 0.090<br>p = 0.597		**Immobility frequency (n)**<br>$r_{(37)}$ = 0.168<br>p = 0.320
			**Grooming frequency (n)**<br>$r_{(37)}$ = 0.142<br>p = 0.402			**Climbing frequency (n)**<br>$r_{(37)}$ = -0.110<br>p = 0.518

Suppl. Table S2 Correlations between dorsal striatum uPA mRNA (upper part) and BDNF protein levels (lower part) and measures of anxiety- and depression-like behaviors.
